# The Occurrence of the Holometabolous Pupal Stage Requires the Interaction between E93, Krüppel-Homolog 1 and Broad-Complex

**DOI:** 10.1371/journal.pgen.1006020

**Published:** 2016-05-02

**Authors:** Enric Ureña, Silvia Chafino, Cristina Manjón, Xavier Franch-Marro, David Martín

**Affiliations:** Institute of Evolutionary Biology (CSIC-Universitat Pompeu Fabra), Barcelona, Spain; New York University, UNITED STATES

## Abstract

Complete metamorphosis (Holometaboly) is a key innovation that underlies the spectacular success of holometabolous insects. Phylogenetic analyses indicate that Holometabola form a monophyletic group that evolved from ancestors exhibiting hemimetabolous development (Hemimetaboly). However, the nature of the changes underlying this crucial transition, including the occurrence of the holometabolan-specific pupal stage, is poorly understood. Using the holometabolous beetle *Tribolium castaneum* as a model insect, here we show that the transient up-regulation of the anti-metamorphic *Krüppel-homolog 1* (*TcKr-h1*) gene at the end of the last larval instar is critical in the formation of the pupa. We find that depletion of this specific *TcKr-h1* peak leads to the precocious up-regulation of the adult-specifier factor *TcE93* and, hence, to a direct transformation of the larva into the adult form, bypassing the pupal stage. Moreover, we also find that the TcKr-h1-dependent repression of *TcE93* is critical to allow the strong up-regulation of *Broad-complex* (*TcBr-C*), a key transcription factor that regulates the correct formation of the pupa in holometabolous insects. Notably, we show that the genetic interaction between *Kr-h1* and *E93* is also present in the penultimate nymphal instar of the hemimetabolous insect *Blattella germanica*, suggesting that the evolution of the pupa has been facilitated by the co-option of regulatory mechanisms present in hemimetabolan metamorphosis. Our findings, therefore, contribute to the molecular understanding of insect metamorphosis, and indicate the evolutionary conservation of the genetic circuitry that controls hemimetabolan and holometabolan metamorphosis, thereby shedding light on the evolution of complete metamorphosis.

## Author Summary

Complete metamorphosis is an evolutionary innovation that has been critical for the success of insects. Phylogenetic relationships reveal that holometabolous insects evolved from ancestors displaying hemimetabolous development. Yet, little is known about the molecular nature of the changes required for such transition, including the evolution of the holometabolan-specific pupal stage. Here, by using *Tribolium castaneum*, we report that the crosstalk between *Krüppel-homolog 1* (*Kr-h1*), *E93* and *Broad-Complex* genes at the end of the larval development has been a key event underlying the formation of the pupa. Interestingly, we show that the interaction between Kr-h1 and E93 is also present in hemimetabolous insects, suggesting that the pupal stage has evolved by the co-option of regulatory mechanisms already present in hemimetabolous insects.

## Introduction

Insects are, by far, the most successful and diversified animal group, with more than two million species described (approximately half of all animal species reported). One of the reasons of this taxonomic richness lies in the appearance of specific novel phenotypic characters known as “key innovations” that has allowed the adaptive radiation of insect species. Several lines of evidence suggest that wings and complete metamorphosis are the two key innovations that have had the most relevant effect on insect diversity through evolution [1,2]. However, whereas the origin, evolution and development of wings have been investigated intensively [3–5], less data is available on the origin and evolution of complete metamorphosis [6–9].

Since their origination from arthropod ancestors, approximately 479 million years ago (Ma) [10], insects have undergone extreme evolution in their postembryonic development, emerging different types of metamorphosis: ametaboly, hemimetaboly and holometaboly [6,11]. The most primitive type is ametaboly, in which immature individuals are miniature versions of the wingless adult form and sexual maturity is achieved through successive molts. In hemimetabolous insects, juvenile (nymphs) and adult forms are very similar and the metamorphosis of the adult-specific structures, the wings and the genitalia, occur during a single stage, the last nymphal instar. Finally, in holometabolous insects the immature larva undergoes a complete morphological transformation to form the adult. The body reorganization is so radical that a two-stage metamorphic process bridged by the holometabolous-specific intermediate pupal stage is required to transform the larva into a winged adult. Despite the relevance of complete metamorphosis in the taxonomic success of Holometabola, the nature of the changes underlying the appearance of the holometabolan pupa remains a puzzling problem in evolutionary and developmental biology.

From an endocrine perspective, the genetic switch between juvenile and adult programs in hemimetabolous and holometabolous insects relies on the same hormone: the sesquiterpenoid juvenile hormone (JH) synthesized by the *corpora allata* glands [12–17]. While JH prevents metamorphosis during the pre-ultimate immature stages, its disappearance in the final juvenile stage allows metamorphosis to occur. The anti-metamorphic effect of JH is mediated by the induction of the C2H2 zinc-finger type transcription factor-encoding gene *krüppel-homolog 1* (*Kr-h1*) [13]. RNAi-mediated knockdown of *Kr-h1* triggers premature adult development in hemimetabolous insects and induces precocious pupation in pre-ultimate instar larvae of holometabolous insects [18–21]. A second important metamorphic gene is *Broad-complex* (*Br-C*), which encodes a member of the bric-a-brac-tramtrack-broad family of transcription factors [22,23]. In contrast to the conserved role of Kr-h1, the functions of *Br-C* have critically changed from hemimetabolous to holometabolous insects. RNAi analysis in the hemimetabolous insects *Oncopeltus fasciatus*, *Pyrrochoris apterus* and *Blattella germanica* revealed that Br-C is specifically required for regulation of wing development, in particular size, shape and vein formation [18,24,25], a function that is conserved in the holometabolous *Tribolium castaneum*, *Drosophila melanogaster* and *Bombyx mori* [23,26–30]. In contrast, Br-C functions in holometabolous insects have expanded to the metamorphic control of pupal commitment, pupal morphogenesis and the inhibition of adult differentiation [26–29,31,32]. In addition to Kr-h1 and Br-C, we have recently described E93 as the conserved master factor that promotes adult metamorphosis in hemimetabolous and holometabolous insects [33]. RNAi-mediated depletion of *E93* prevents adult metamorphosis and induces endless repetitions of nymphal molts in hemimetabolous insects and the repetition of the pupal program in holometabolous insects [33]. In addition, E93 is also required to repress the expression of *Kr-h1* and *Br-C* during the last immature stages of hemimetabolous and holometabolous insects, thus ensuring the transition to the adult forms [33].

Given the functional relevance of *Kr-h1*, *E93* and *Br-C* genes (hereafter referred to as the *metamorphic genetic network*), we hypothesized that the appearance of the holometabolan-specific pupal stage was facilitated by changes in the timing of expression and/or regulation of the metamorphic network genes from hemimetabolous to holometabolous insects. In this study, we use the holometabolous insects *Tribolium castaneum* and *Drosophila melanogaster* to test this hypothesis. Here, we show that a transient peak of *Kr-h1* at the end of the final larval stage, a particular event specific of holometabolous insects, has been critical for the occurrence of the pupa. This late pulse of *Kr-h1* prevents the precocious up-regulation of *E93* during this stage, thus pausing the implementation of the adult differentiation program initiated at the prepupal stage, and allowing the strong up-regulation of *Br-C*, which is critical for the correct formation of the pupa. In addition, we use the hemimetabolous insect *Blattella germanica* to demonstrate that the functional relation between Kr-h1 and E93 is evolutionary conserved, thus suggesting that the occurrence of the pupal stage has been facilitated by the co-option of regulatory mechanisms already present in hemimetabolous insects.

## Results

### *TcKr-h1* prevents direct adult differentiation during the prepupal stage of *T*. *castaneum*

**Fig 1:**
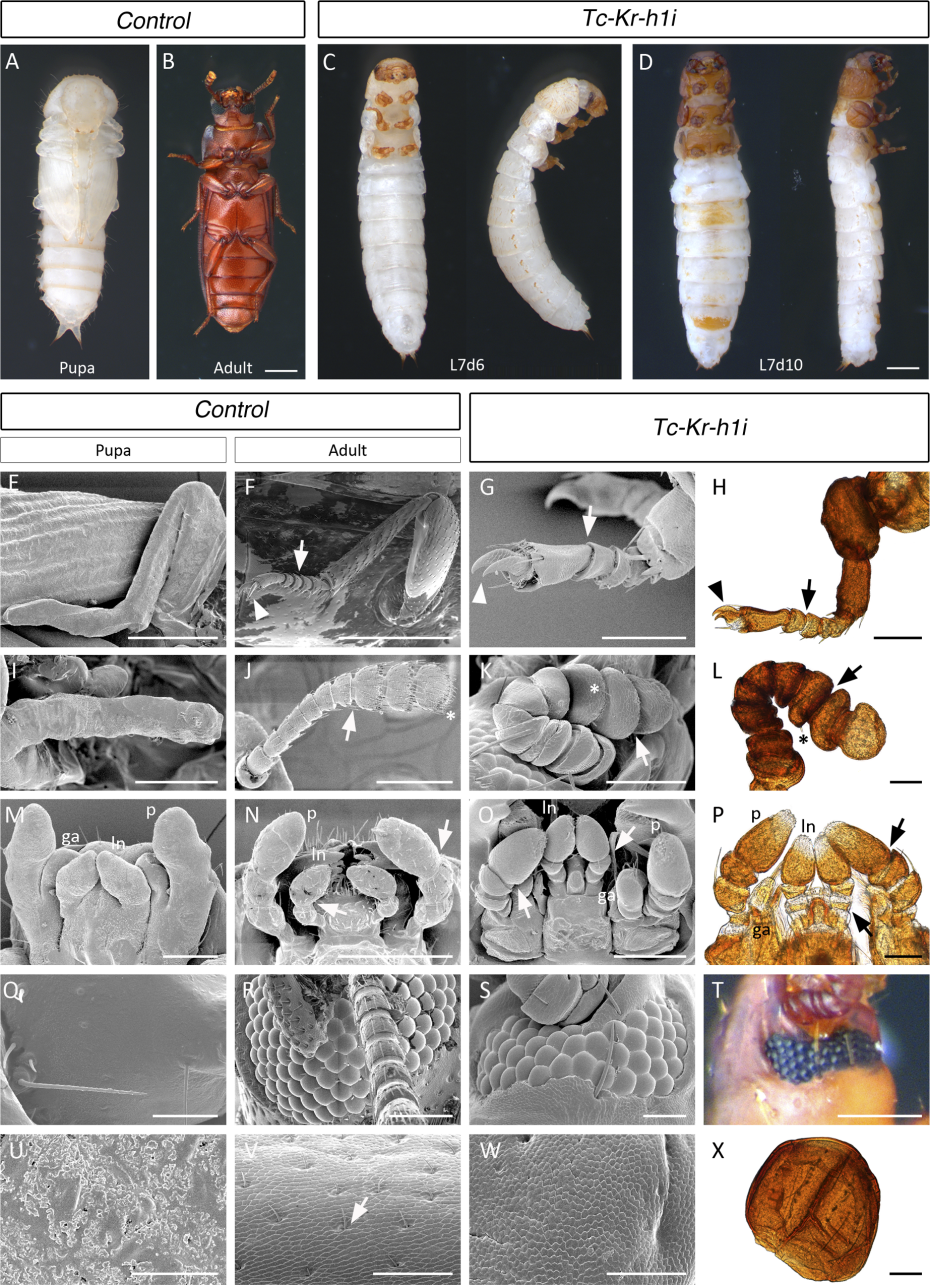
Depletion of *TcKr-h1* in *T*. *castaneum* last instar larvae causes direct transformation to the adult form bypassing the pupal stage. (*A-D*) Newly molted L7 larvae were injected with *dsMock* (*Control*) or with *dsTcKr-h1* (*TcKr-h1i*). Ventral views of (*A*) a *Control* pupa, and (*B*) a *Control* adult. Ventral and lateral views of (*C*) 6- and (*D*) 10-day-old *TcKr-h1i* animals. (*E-X*) Comparison of the external morphology of appendages between *Control* pupae and adults, and *TcKr-h1i* animals. Scanning electron microscopy photographs of (*E*, *I*, *M*, *Q* and *U*) a newly molted *Control* pupa, (*F*, *J*, *N*, *R* and *V*) a 1-day-old *Control* adult, and (*G*, *K*, *O*, *S* and *W*) a 6-day-old *TcKr-h1i* animal, showing strong direct adult differentiation in *TcKr-h1i* animals. (*G*) *TcKr-h1i* legs are clearly segmented, including the tarsus (arrow), and presents double claws typical for the adult leg (arrowhead). (*K*) *TcKr-h1i* antennae show adult sensillae (asterisks) with well-shaped and segmented funicle and club (arrow). (*O*) All *TcKr-h1i* mouthparts are strongly segmented (arrows), including the (ln) labial palps, (p) maxillary palps and (ga) galea. (*S*) *TcKr-h1i* compound eyes present several rows of well-developed ommatidia. (*W*) Elytra of *TcKr-h1i* animals are highly sclerotized with the typical adult microsculpture. (*H*, *L*, *P*, *T* and *X*) External morphology of the appendages of *TcKr-h1i* animals showing that they are highly sclerotized and present uniformly dark brown pigmentation, characteristic of adult appendages.

**Fig 2:**
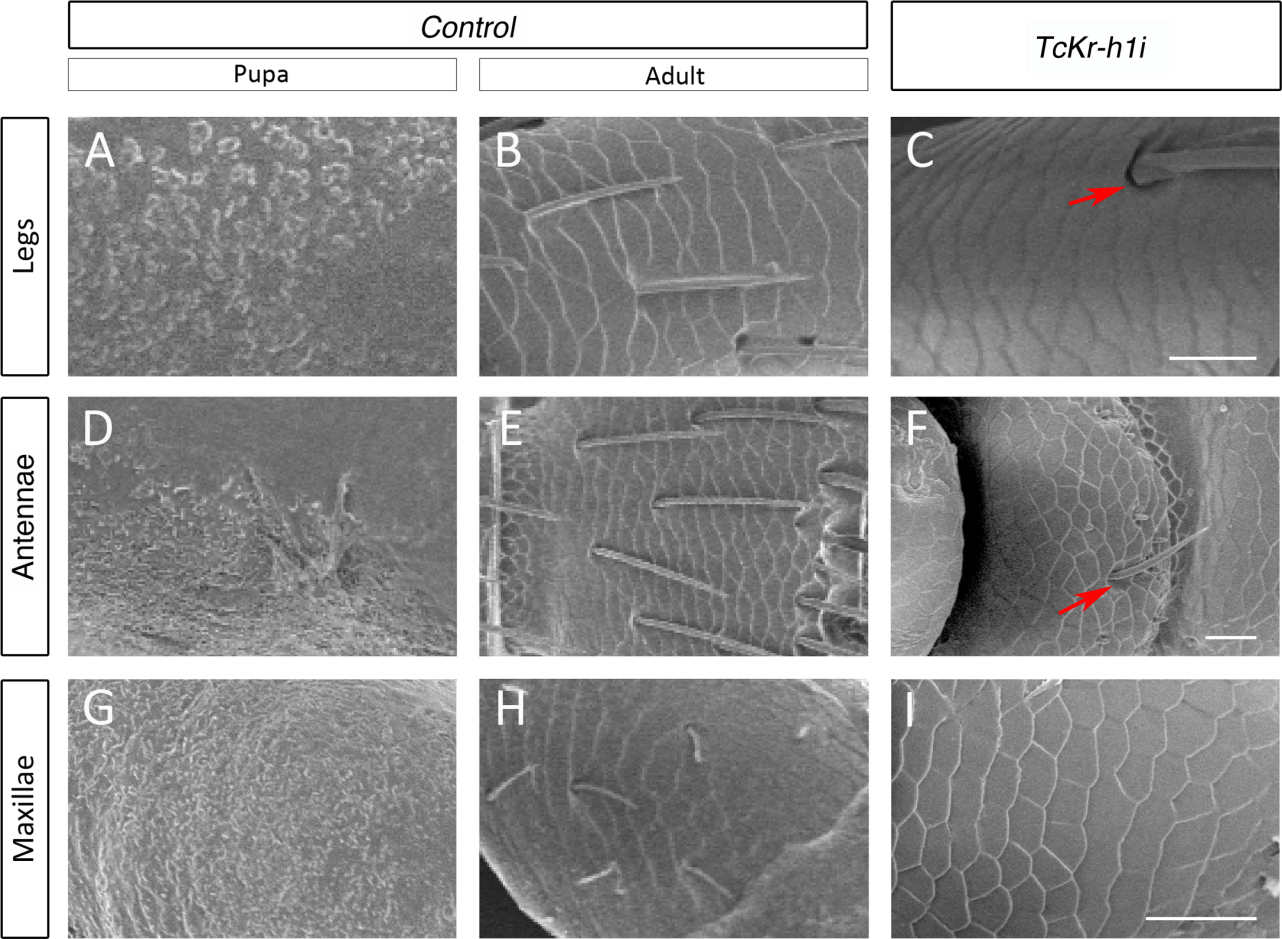
The cuticle of the appendages of *TcKr-h1*-depleted animals shows adult-specific microsculpture. Comparison of scanning electron microscopy photographs of the cuticle surface in (*A-C*) legs, (*D-F*) antennae, and (*G-I*) maxillae of (*A*, *D* and *G*) *Control* pupae, (*B*, *E* and *H*) *Control* adults, and (*C*, *F* and *I*) *TcKr-h1i* animals. Instead of the pupal-like surface, the cuticle of *TcKr-h1i* animals shows the characteristic adult-specific microsculpture, including rounded pits with sensillae (arrows in *C* and *F*). Scale bars represent 50 μm in (*C*), (*F*) and (*I*).

**Fig 3:**
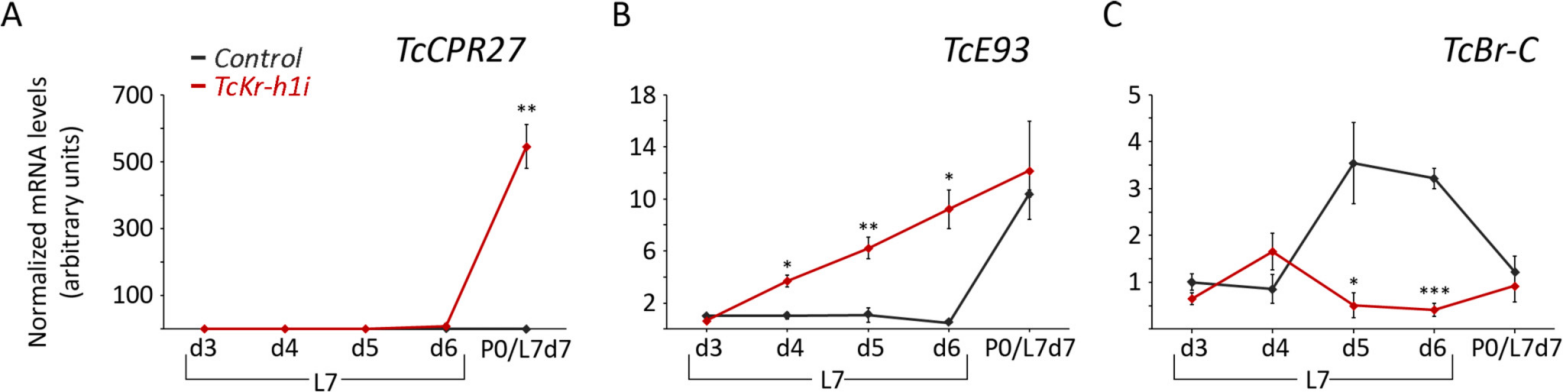
Loss of *TcKr-h1* in last instar larvae induces precocious up-regulation of the adult specifier *TcE93* and the repression of the pupal specifier *TcBr-C*. (*A-C*) Transcript levels of (*A*) the adult-specific gene *TcCPR27*, (*B*) *TcE93*, and (*C*) *TcBr-C* were measured by qRT-PCR in *Control* and *TcKr-h1i* animals. Transcript abundance values are normalized against the *TcRpL32* transcript. Fold changes are relative to the expression of each gene in 3-day-old *Control* larvae, arbitrarily set to 1. In the abscissa axis, P0/L7d7 represents equivalent developmental time points between *Control* (P0, pupal day 0) and *TcKr-h1i* animals (L7d7, day 7 in arrested *TcKr-h1i* animals). Error bars indicate the SEM (n = 5). Asterisks indicate differences statistically significant at p≤0.05 (*); p≤0.005 (**), and p≤0.0005 (***) (*t*-test).

Three events are critical to promote metamorphosis in last instar nymphs of hemimetabolous insects: (i) the drop in the JH titer, (ii) the down-regulation of the anti-metamorphic factor *Kr-h1*, and (iii) the up-regulation of *E93*, which induces adult differentiation [33]. Likewise, these three events also occur during the onset of the last larval instar of the holometabolous *T*. *castaneum* (S1 Fig) [20,33]. However, a distinguishing event in *T*. *castaneum* is the up-regulation of *TcKr-h1* expression during the prepupal stage at the end of the final (L7) larval instar (S1 Fig), suggesting that this *Kr-h1* prepupal elevation might be a key event in the evolution of complete metamorphosis. Although the role of TcKr-h1 in pre-ultimate larval stages of *T*. *castaneum* has been analyzed previously [20], its specific function during the prepupal stage is unknown. Thus, to examine the function of the late larval peak of *TcKr-h1* expression, we depleted *TcKr-h1* by RNAi *in vivo* by injecting *TcKr-h1* dsRNA into newly emerged last L7 instar larvae (*TcKr-h1i* animals). Specimens injected with *dsMock* were used as negative controls (*Control* animals). Whereas *Control* animals pupated normally, the majority of the *TcKr-h1i* larvae arrested development after six days of injection (S1 Table). Remarkably, removal of the apolysed larval cuticle from the arrested *TcKr-h1i* larvae revealed a strong precocious adult development, especially in the head and thorax (Fig 1A–1D). For example, in the head several rows of ommatidia were clearly developed in the compound eyes of *TcKr-h1i* animals (Fig 1Q–1S). The antennae presented adult sensillae and the shape and segmentation of the funicle and club were clearly adult-like (Fig 1I–1K). The maxillae showed well-defined and segmented palps, lacinia and galea (Fig 1M–1O). In the thorax, the legs presented the double claws typical for the adult legs and the different segments, including the tarsal ones, were clearly defined (Fig 1E–1G). The elytra were highly sclerotized with the cuticle showing the typical adult microsculpture (Fig 1U–1W). The adult-specific microsculpture was also detected in all body appendages (Fig 2). Furthermore, the dark brown pigmentation of the tanned cuticle in the head and thorax, including the appendages and part of the abdomen, resembled that of the adult (Fig 1H, 1L, 1P, 1T and 1X). On the other hand, the abdomen of *TcKr-h1i* animals showed less pronounced adult differentiation and several pupal structures, such as the gin traps and urogomphi, were also visible (S2 Fig). Confirming the premature activation of the adult genetic program in *TcKr-h1i* animals, high levels of the adult-specific cuticle gene *TcCPR27*, normally occurring at the end of the pupal stage [34], were precociously detected at the end of the larval stage (Fig 3A). Overall, our data demonstrate that the specific up-regulation of *TcKr-h1* at the end of larval development prevents the larva to metamorphose directly into the adult bypassing the pupal stage.

Scale bars represent 0.5 mm in (*B*) and (*D*); 300 μm in (*E*) and (*F*); 200 μm in (*I*), (*J*), (*N*) and (*T*); 100 μm in (*G*), (*H*), (*K*), (*M*), (*O*), (*R*) and (*X*); 50 μm in (*L*), (*P*), (*Q*), (*S*), (*V*) and (*W*); 30 μm in (*U*).

### TcKr-h1 blocks direct adult differentiation by repressing *TcE93* at the larval-pupal transition

In order to understand how the prepupal pulse of *TcKr-h1* prevents direct adult differentiation, we next analyzed the expression of *TcBr-C* and *TcE93* in *TcKr-h1i* animals, as these factors are required for the correct formation of the pupal and adult forms, respectively [26–28,33]. In wild type *T*. *castaneum*, *TcE93* mRNA levels start to increase moderately in the last larval instar to reach maximum levels during the pupal stage, while *TcBr-C* expression is restricted to a strong pulse during the prepupal stage (S1 Fig) [26–28,33]. Consistent with the precocious differentiation of adult features in *TcKr-h1i* animals, we found that *TcE93* mRNA levels were prematurely up-regulated in these animals and that the prepupal peak of *TcBr-C* mRNA was strongly suppressed (Fig 3B and 3C). These results suggest that the direct transition from larva to adult in *TcKr-h1i* animals stems from the premature up-regulation of *TcE93* at the end of L7 and the concomitant repression of *TcBr-C*. In light of our previous study showing that TcE93 represses *TcBr-C* expression in the pupal stage [33], our findings also suggest that the repression of *TcBr-C* in the *TcKr-h1i* animals may depend on the untimely increase of *TcE93*.

**Fig 4:**
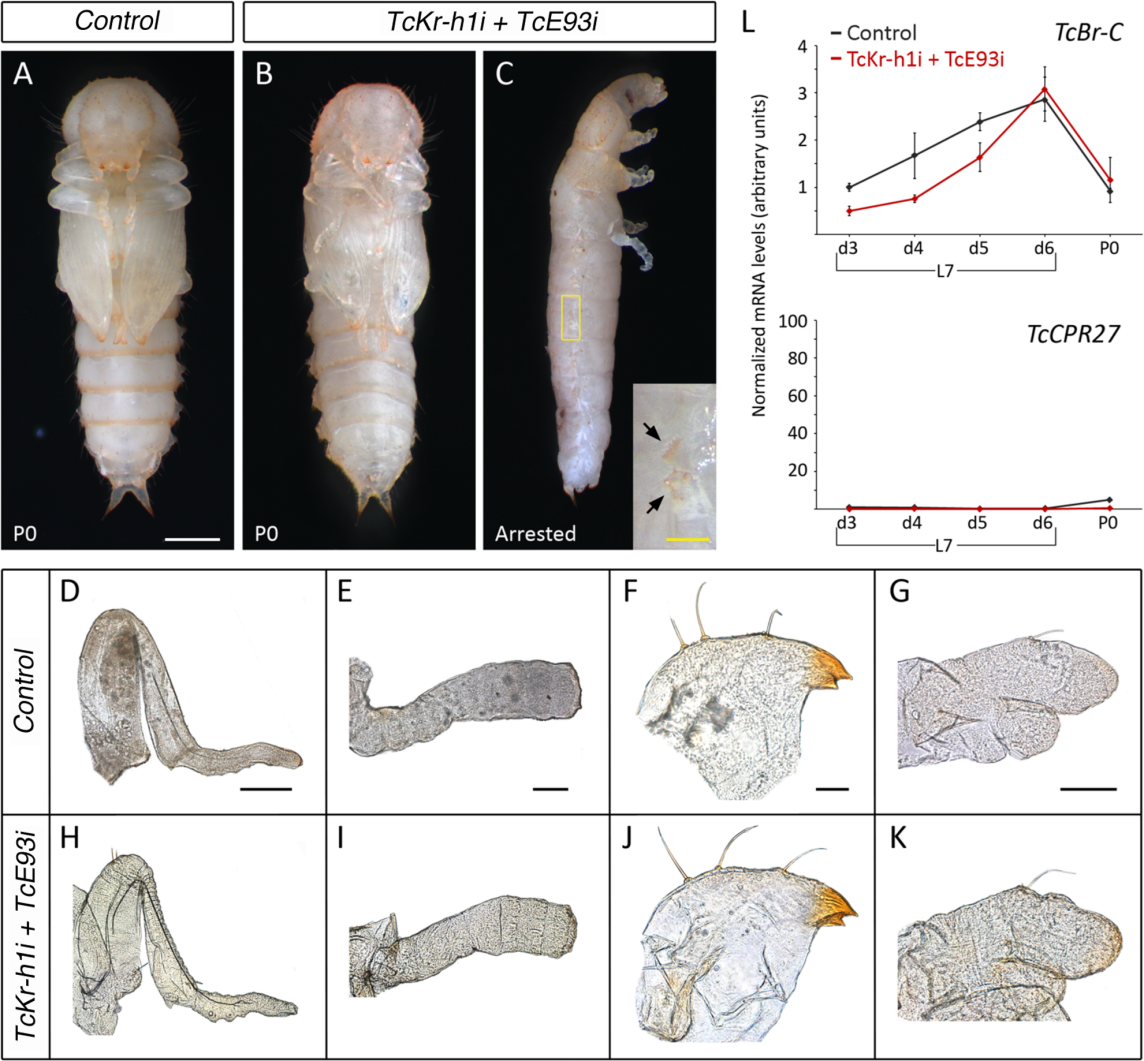
Precocious high levels of TcE93 mediate the direct adult transformation of *TcKr-h1*-depleted larvae and prevent the up-regulation of *TcBr-C*. (*A-C*) Newly molted L7 larvae were injected with *dsMock* (*Control*) or with *dsTcKr-h1* and *dsTcE93* simultaneously (*TcKr-h1i + TcE93i*). (*A*) Ventral view of a *Control* pupa. (*B*) Ventral and (*C*) lateral views of *TcKr-h1i + TcE93i* knockdown animals. In *C*, the *TcKr-h1i + TcE93i* animal arrested development and could not undergo pupal ecdysis, so the larval cuticle has been removed. The inset is a high magnification of the abdomen of the arrested *TcKr-h1i + TcE93i* pupa (rectangle in *C*) showing the pupal gin traps (arrows). (*D-K*) Comparison of the external morphology of appendages between (*D-G*) *Control*, and (*H-K*) *TcKr-h1i + TcE93i* pupae. *TcKr-h1i + TcE93i* pupae undergo normal pupation as no differences in appendage morphology are observed when compared to *Control* animals. (*D* and *H*) Hindlegs, (*E* and *I*) antennae, (*F* and *J*) mandible, and (*G* and *K*) maxilla. (*L*) Simultaneous depletion of *TcKr-h1* and *TcE93* leads to normal expression of *TcBr-C* and *TcCPR27* in *T*. *castaneum* prepupae. *TcBr-C* (upper), and *TcCPR27* (lower) mRNA levels were measured by qRT-PCR in *Control* and *TcKr-h1i + TcE93i* animals. Transcript abundance values are normalized against the *TcRpL32* transcript. Fold changes are relative to the expression of each gene in 3-day-old *Control* larvae, arbitrarily set to 1. Error bars indicate the SEM (n = 5). Scale bars: 0.5 mm in (*A*); inset in (*C*), 0.1 mm; 200 μm in (*D*); 100 μm in (*E*) and (*G*); 50 μm in (*F*).

If both suggestions were correct, then depleting *TcKr-h1* and *TcE93* simultaneously in L7 would be sufficient to impair premature adult differentiation and to allow the normal induction of *TcBr-C*, thus redirecting the molt again to a normal pupal stage. Consequently, we injected *TcKr-h1* and *TcE93* dsRNAs into newly emerged last L7 instar larvae (*TcKr-h1i*+*TcE93i* animals). Most of the *TcKr-h1i*+*TcE93i* animals pupated properly 6 days after the injection (Fig 4A and 4B, S2 Table). Remarkably, all the appendages of these animals showed normal pupal-like morphology with no signs of precocious adult differentiation (Fig 4A and 4B). In addition, some of the double knockdown animals arrested development at the prepupal-pupal transition (Fig 4C and S2 Table). When the larval cuticle of these animals was removed, the morphology of the appendages was similar to those that had successfully pupated, including well-developed gin traps in the abdomen (Fig 4C–4K). Consistent with the observed phenotype, *TcKr-h1i*+*TcE93i* prepupae presented a normal peak of *TcBr-C* mRNA and the premature up-regulation of the adult-specific *TcCPR27* gene was completely prevented (Fig 4L). Altogether, our results show that the transient pulse of *TcKr-h1* at the end of the larval development prevents the direct transformation of larval tissues to adult ones by maintaining low levels of *TcE93* during this period. In doing so, TcKr-h1 allows the strong up-regulation of *TcBr-C* and the occurrence of a new developmental stage, the pupa.

**Fig 5:**
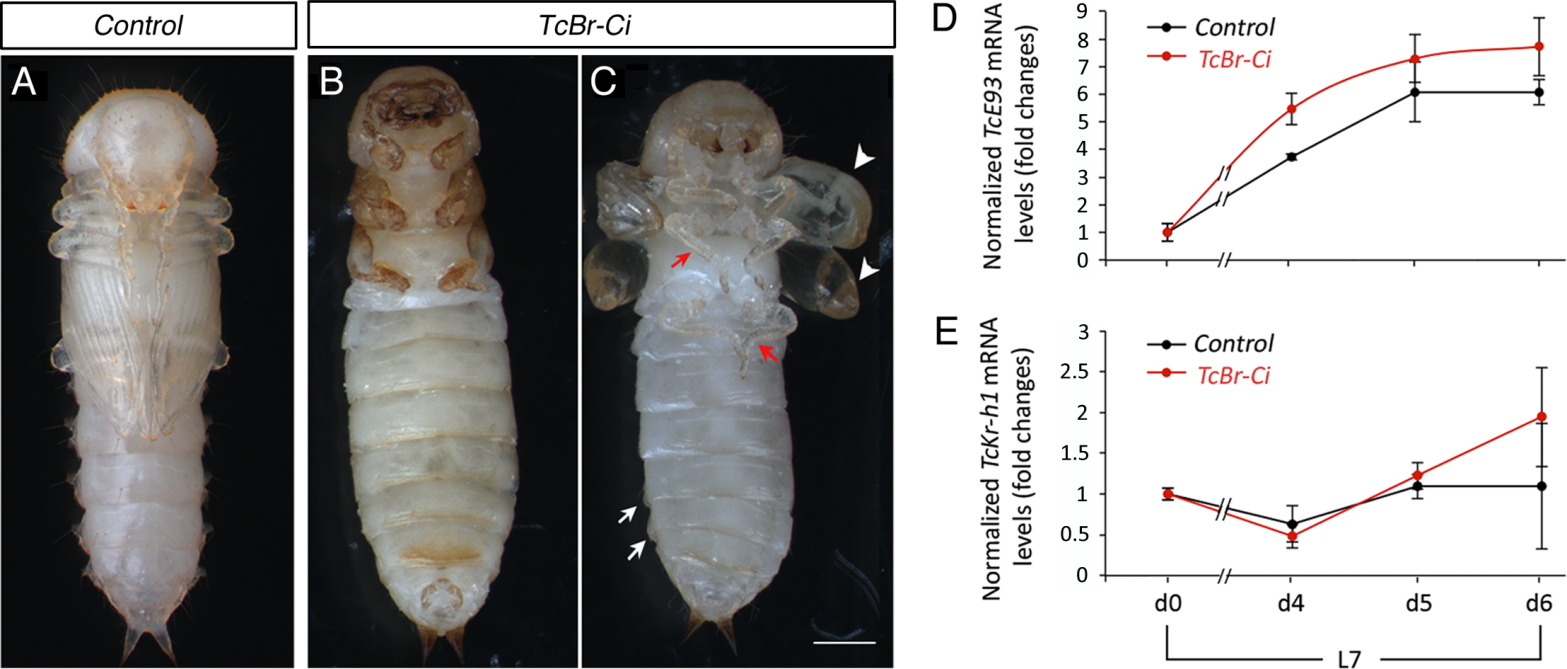
Loss of *TcBr-C* induces premature adult differentiation without affecting *TcE93* and *TcKr-h1* expression. (*A*-*C*) Newly molted L7 larvae were injected with *dsMock* (*Control*) or with *dsTcBr-C* (*TcBr-Ci*). (*A*) Ventral view of a *Control* pupa. (*B* and C) Ventral views of (B) a *TcBr-Ci* animal arrested at the end of the L7 stage, and (C) a *TcBr-Ci* animal just after the pupal molt, showing abnormal shape with short and blister wings (arrowheads), imperfect gin traps (arrows) and short legs (red arrows). (*D* and *E*) Transcript levels of (*D*) *TcE93*, and (*E*) *TcKr-h1* in *Control* and *TcBr-Ci* larvae at the beginning of the last L7 instar (d0) and during the quiescent stage (d4-d6), measured by qRT-PCR. Transcript abundance values are normalized against the *TcRpL32* transcript. Fold changes are relative to the expression of each gene in d0 *Control* larvae, arbitrarily set to 1. Error bars indicate the SEM (n = 5–10). Scale bar: 0.5 mm.

Next, we asked to what extent the adult features observed in *TcKr-h1i* animals, consequence of the premature *TcE93* up-regulation, were due to the TcE93-dependent repression of *TcBr-C*. To this aim, we injected *TcBr-C* dsRNA in newly molted L7 larvae (*TcBr-Ci* animals) to mimic the absence of the prepupal *TcBr-C* peak observed in *TcKr-h1i* animals. Consistent with previous reports [26–28], the majority of the *TcBr-Ci* larvae arrested development at the end of the prepupal stage or just after the pupal molt showing a mix of larval, pupal and adult characters (Fig 5A–5C and S3 Table). Detailed analyses of the *TcBr-Ci* specimens that undergo pupation revealed that, in addition to short and blister wings, abnormal urogomphi and absence of gin traps, their appendages, including antennae, maxillae, mandibles, and legs, presented an adult-like segmentation although with larval-like pigmentation (S3 Fig; [26–28]). However, the extent of adult differentiation of *TcBr-Ci* animals was clearly weaker than that observed after *TcKr-h1* removal (Fig 1), suggesting that the premature adult differentiation observed in *TcKr-h1i* specimens was not exclusively channeled through *TcBr-C* repression. We, then, measured the expression levels of *TcE93* and *TcKr-h1* in *TcBr-Ci* animals during the prepupal stage and found that the mRNA levels of both genes were similar to *Control* animals (Fig 5D and 5E), indicating that phenotypic alterations observed in *TcBr-Ci* animals are not due to variations of *TcE93* or *TcKr-h1* levels. Taken together, our data suggest that high levels of *TcBr-C*, together with low levels of *TcE93*, which depend of the *TcKr-h1* peak, are required during the prepupal stage of *T*. *castaneum* to prevent premature adult differentiation and allow the proper formation of the pupa.

### The interplay between Kr-h1, E93 and Br-C is conserved in the derived holometabolous *Drosophila melanogaster*

**Fig 6:**
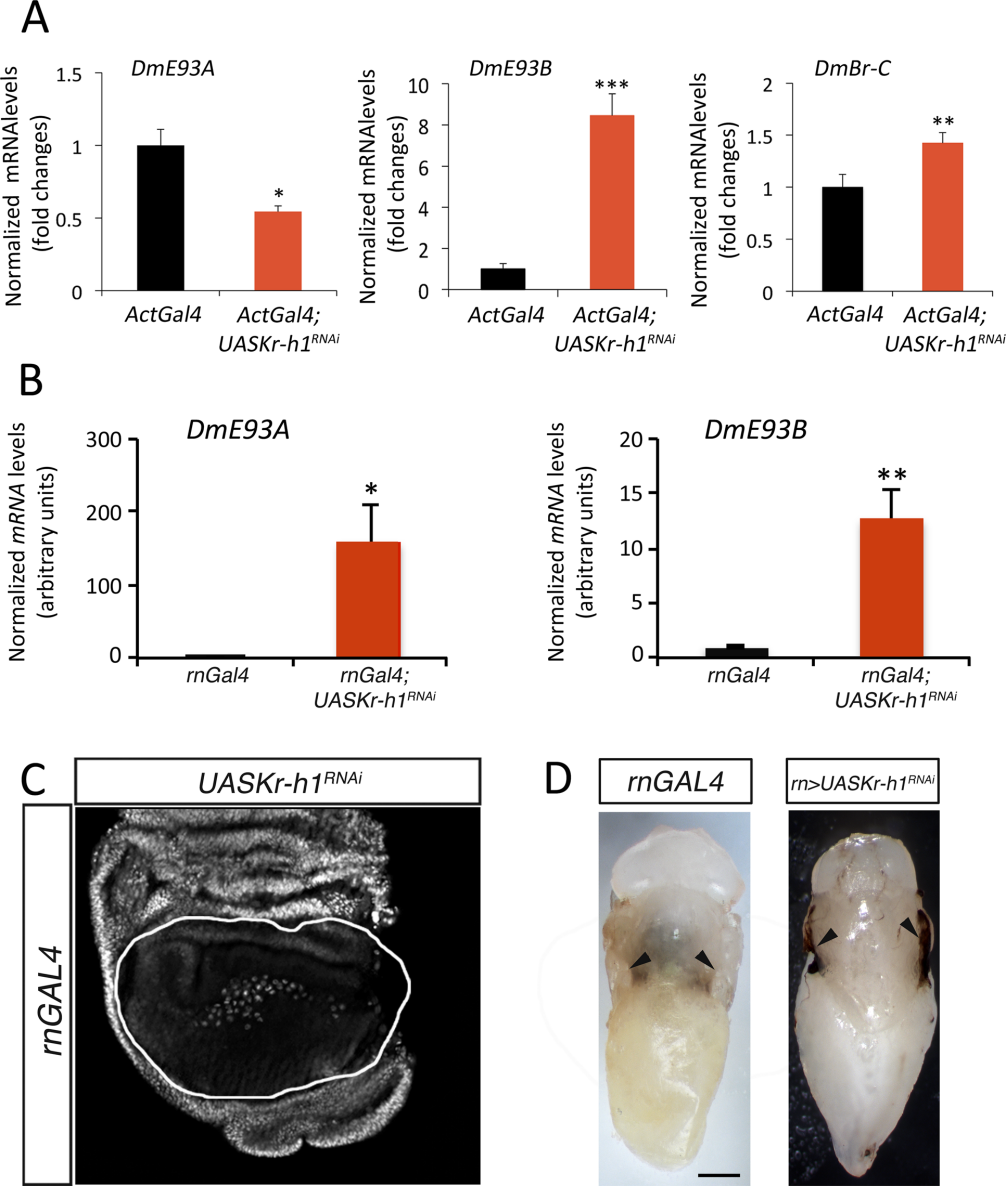
The crosstalk between the metamorphic network genes is conserved in the derived holometabolous insect *D*. *melanogaster*. (*A*) Expression levels of *DmE93A* (left), *DmE93B* (middle), and *DmBr-C* (right) relative to *DmRpL32* in the whole body of *ActGal4* (in black) and *ActG*al4;*UASDmKr-h1*^*RNAi*^ (in red) 0 h after puparium formation (APF) animals, measured by qRT-PCR. Fold changes are relative to the expression of each gene in *ActGal4* larvae, arbitrarily set to 1. (*B*) Expression levels of *DmE93A* (left) and *DmE93B* (right) relative to *DmRpL32* specifically in the wing pouch of *rnGal4* (in black) and *rn*Gal4;*UASDmKr-h1*^*RNAi*^ (in red) 0 h after puparium formation (APF) animals, measured by qRT-PCR. Fold changes are relative to the expression of each gene in *rnGal4* larvae, arbitrarily set to 1. Error bars in A and B indicate the SEM (n = 5–10). Asterisks indicate differences statistically significant at p≤0.05 (*), p≤0.005 (**), and p≤0.0005 (***) (*t*-test). (*C*) DmBr-C protein levels revealed by immunocytochemistry in wings from 0 h after puparium formation (APF) larvae expressing a *UASDmKr-h1*^*RNAi*^ constructs under the control of *rnGal4* driver, which is expressed in the wing pouch (silhouetted). DmBr-C protein is absent in *DmKr-h1*-depleted cells. (*D*) Dorsal views of *rnGAL4* (left panel) and *rn*Gal4;*UASDmKr-h1*^*RNAi*^ (right panel) pupae. In the absence of *DmKr-h1* in the wing pouch, wings degenerate after eversion during the pupal stage (arrowheads). Scale bars: 200 μm in (*D*).

**Fig 7:**
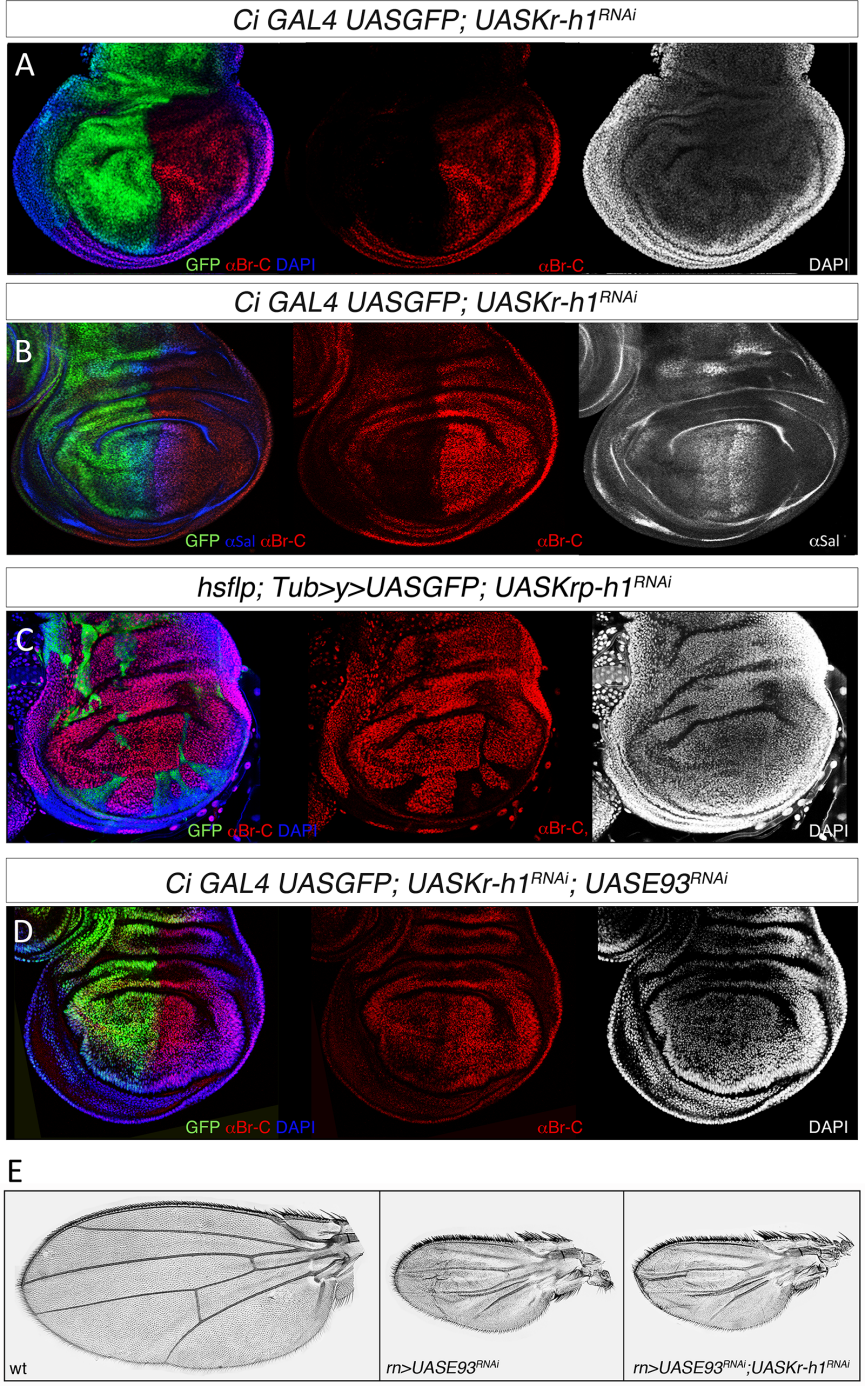
DmBr-C expression is repressed by DmE93 in the prepupal stage of *D*. *melanogaster*. (*A*) Representative wing disc of *CiGal4 UASGFP; UASKr-h1*^*RNAi*^ 0 h APF prepupae labelled to visualize the Ci anterior domain (GFP in green), the nuclei (DAPI) and DmBr-C protein (in red). In the Ci domain, where the cells express the transgene and are depleted of *DmKr-h1*, *DmBr-C* is strongly repressed. (*B*) Representative wing disc of *CiGal4 UASGFP; UASKr-h1*^*RNAi*^ 0 h APF prepupae labelled to visualize the Ci anterior domain (GFP in green), DmBr-C protein (in red), and the transcription factor Spalt (in white). Whereas DmBr-C protein levels are strongly reduced in the Ci domain, normal levels of Spalt protein are present in the anterior and posterior domains of the wing disc indicating that the viability of the *DmKr-h1-*depleted cells is not compromised. (*C*) Examples of clones of cells in *TubUASGFP; UASDmKr-h1*^*RNAi*^ wing discs. Wings discs are labeled to visualize the clones (GFP in green), the nuclei (DAPI) and DmBr-C protein (in red). DmBr-C protein in cells within the clones is absent. (*D*) Representative wing disc of *CiGal4 UASGFP; UASKr-h1*^*RNAi*^ *UASDmE93*^*RNAi*^ 0 h APF prepupae labelled to visualize the Ci anterior domain (GFP in green), the nuclei (DAPI) and DmBr-C protein (in red). In the Ci domain, where the cells are depleted of *DmKr-h1* and *DmE93*, DmBr-C protein levels are not reduced. (*E*) Cuticle preparations of adult wings expressing the indicated transgenes under the control of the *rnGAL4* driver.

Next, we investigated whether the metamorphic genetic circuitry is conserved in more derived holometabolous insects and turned to the dipteran *D*. *melanogaster*. To this end, we used the *Gal4/UAS* system [35] to modify the expression of the metamorphic genes. First, we knocked down *DmKr-h1* expression in the whole animal using the *UAS-DmKr-h1*^*RNAi*^ transgene driven by the ubiquitous *ActinGal4 (ActGal4)* driver, and measured the levels of *DmE93* and *DmBr-C* in white prepupa. Similar to *T*. *castaneum*, we found that depletion of *DmKr-h1* lead to a significant increase of the mRNA levels of the two *DmE93* isoforms, *DmE93A* and *DmE93B*, and to the concomitant decrease of *DmBr-C* mRNA levels (Fig 6A). To confirm this result specifically in a metamorphic tissue, we overexpressed *UAS-DmKr-h1*^*RNAi*^ specifically in the pouch region of the wing disc using the *rotundGal4 (rnGAL4*) driver. As expected, depletion of *DmKr-h1* led to a remarkable increase of *DmE93A* and *DmE93B* in the wing pouch during the prepupal stage (Fig 6B), and to the disappearance of DmBr-C protein (Fig 6C). However, unlike *T*. *castaneum* where the viability of the wings was not affected (S4 Fig), *DmKr-h1*-depleted wings showed clear signs of necrosis at the pupal stage (Fig 6D), probably due to a deficient wing evertion. The wing phenotype is consistent with previous reports where imaginal discs failed to elongate properly in *D*. *melanogaster Br-C* mutants [23,30,36]. To further confirm the repression of *DmBr-C*, at the protein level, *UAS-DmKr-h1*^*RNAi*^ was specifically expressed in the anterior compartment of the wing disc by using the *cubitus interruptusGAL4* (*CiGal4UASGFP*) driver, and its effect there was compared with the control posterior compartment. As Fig 7A shows, depletion of *DmKr-h1* with *CiGal4UASGFP* drastically reduced DmBr-C protein levels in the anterior compartment. The disappearance of DmBr-C was not due to reduced viability of the *DmKr-h1*-depleted cells as they showed normal protein levels of Spalt, a protein whose expression is independent of either *DmKr-h1* or *DmE93* (Fig 7B). The same result was obtained when *DmKr-h1*^*RNAi*^ clones were generated in the wing disc (Fig 7C), confirming again the cell autonomous repression of *DmBr-C*.

Next, we analyzed whether the disappearance of DmBr-C was due to the precocious upregulation of *DmE93*. To do this, we depleted both *DmKr-h1* and *DmE93* simultaneously in the anterior compartment of the wing pouch using the *CiGal4UASGFP* driver. Under these conditions, the levels of DmBr-C protein returned to normal (Fig 7D), allowing the normal evertion of the wing disc. Likewise, when *DmKr-h1* and *DmE93* were depleted in the wing pouch under the control of the *rnGal4* driver, the wings did not degenerate and everted properly, and the adult flies emerged with small and undifferentiated wings, a phenotype similar to that observed in *DmE93*-depleted adult wings (Fig 7E). Altogether, our results show that the regulatory interactions between the metamorphic toolkit genes are conserved in more derived holometabolous insects.

### The anti-metamorphic effect of Kr-h1 is functionally channeled through E93 repression also in hemimetabolous insects

**Fig 8:**
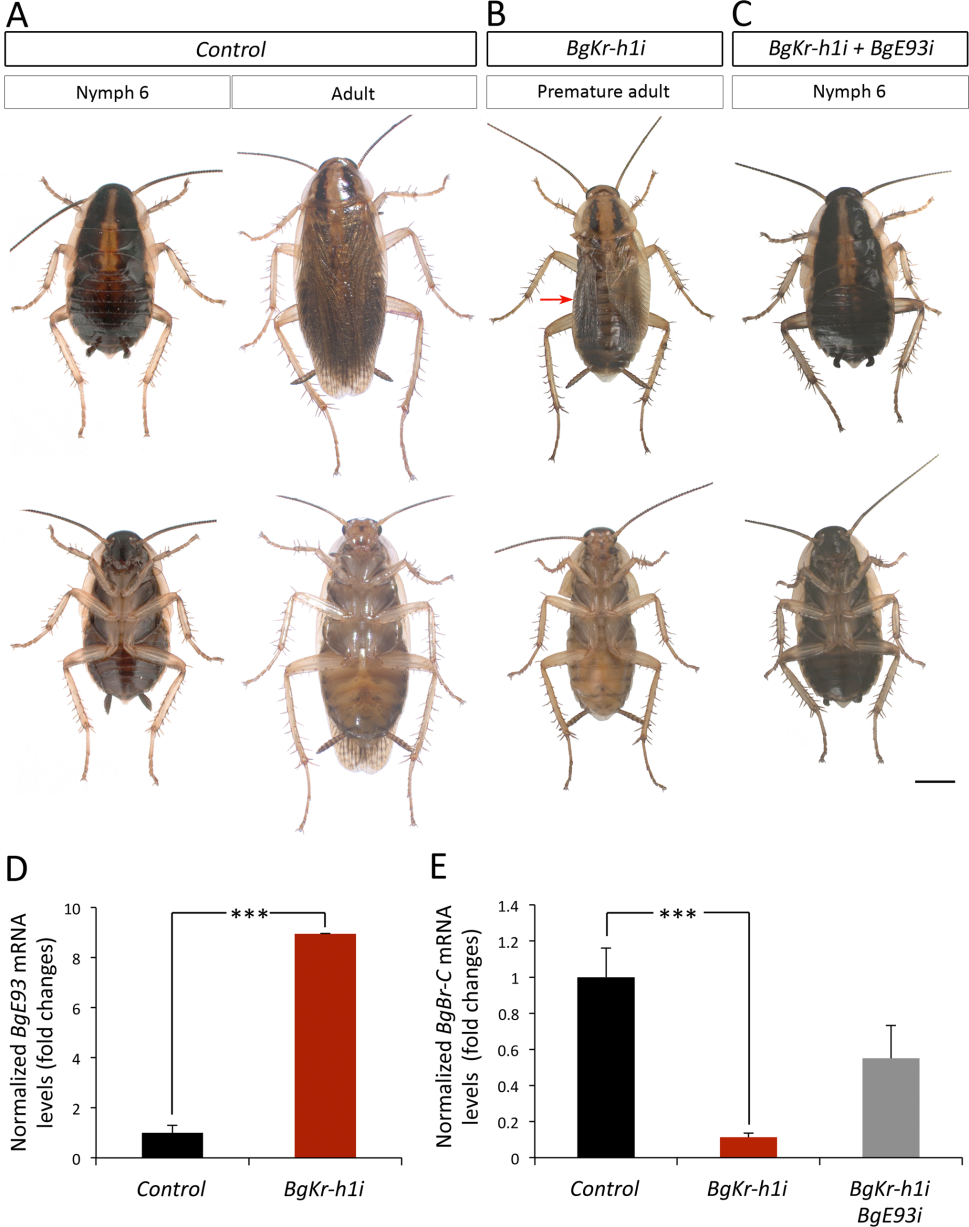
The Kr-h1-dependent repression of adult differentiation is relayed through E93 repression also in hemimetabolous insects. (*A-C*) Newly molted N5 nymphs of *B*. *germanica* were injected with *dsMock* (*Control*), with *dsBgKr-h1* (*BgKr-h1i*) or with *dsBgK-h1* and *dsBgE93* simultaneously (*BgKr-h1i + BgE93i*) and left until the next molts. (*A*) Dorsal (upper panels) and ventral (lower panels) views of a N6 *Control* nymph and a winged adult. (*B*) Dorsal and ventral views of a *BgKr-h1i* animal after the next molt showing a premature adult morphology, including functional hindwings and forewings, adult-specific pigmentation of the cuticle and the pronotum, and adult cerci. In the dorsal view, the left forewing has been removed to allow the observation of the membranous hindwing (red arrow). (*C*) Dorsal and ventral views of a *BgKr-h1i + BgE93i* animal after the next molt showing a perfect N6 morphology that includes black cuticle, two thick stripes of black melanin in the pronotum, nymphal cerci and external wing pads. (*D* and *E*) Loss of *BgKr-h1* during N5 induces (*D*) precocious up-regulation of *BgE93*, and (*E*) repression of *BgBr-C* in N5 nymphs. The repression of *BgBr-C* is averted when *BgKr-h1* and *BgE93* are simultaneously depleted in N5 nymphs. Transcript levels of (*D*) *BgE93*, and (*E*) *BgBr-C* were measured by qRT-PCR in wings from 5-day-old *Control*, *BgKr-h1i* and *BgKr-h1i*+*BgE93i* N5 nymphs. Transcript abundance values are normalized against the *BgActin5C* transcript. Fold changes are relative to the expression of each gene in *Control* nymphs, arbitrarily set to 1. Error bars indicate the SEM (n = 5–10). Asterisks indicate differences statistically significant at p≤0.0005 (***) (*t*-test). Scale bar represents 2 mm.

Given that Holometaboly evolved from hemimetabolous ancestors some 345 Ma [10], we finally sought to determine whether the effect of Kr-h1 in preventing precocious adult metamorphosis through E93 is present also in hemimetabolous insects. To this end, we turned to the German cockroach *Blattella germanica* as a model of hemimetabolous development. *B*. *germanica* undergoes six nymphal instars (N1-N6) before molting into the adult. In contrast to holometabolous insects, *B*. *germanica* metamorphosis occurs during a single period, the last (N6) nymphal instar, and is restricted to the transformation of the wing primordia into functional wings, to the acquisition of functional genitalia and to marked changes in cuticle pigmentation [33,37]. In agreement with previous data [19], RNAi-mediated depletion of *BgKr-h1* in penultimate (N5) instar nymphs (*BgKr-h1i* animals) caused precocious differentiation of adult features after the ensuing molt (Fig 8A and 8B, S4 Table). Precocious *BgKr-h1i* adults were smaller but had all the external characteristics of a normal adult: functional hind- and forewings, adult cerci, and adult-specific pigmentation of the cuticle (Fig 8B). Consistent with the phenotype observed, *BgKr-h1i* N5 nymphs presented a significant precocious up-regulation of *BgE93* when compared to *Control* nymphs (Fig 8D). This result was consistent with a previous report [38], although in their experiments, *BgE93* levels were measured specifically in the tergal gland of male nymphs. Interestingly, as happened in holometabolous insects, we also found that *BgBr-C* mRNA levels were strongly reduced in *BgKr-h1i* N5 nymphs (Fig 8E).

To confirm that the premature activation of the adult genetic program in *BgKr-h1i* animals depends on the precocious up-regulation of BgE93, as in *T*. *castaneum TcKr-h1i* prepupae, we next depleted *BgKr-h1* and *BgE93* simultaneously in penultimate N5 nymphs. As expected, double RNAi for *BgKr-h1* and *BgE93* (*BgKr-h1i+BgE93i* animals) in newly emerged N5 nymphs resulted in normal N6 nymphs after the following molt instead of undergoing precocious metamorphosis (Fig 8C and S4 Table). *BgKr-h1i+BgE93i* N6 nymphs presented all the morphological characteristics of a nymph: black cuticle, two thick stripes of black melanin in the pronotum, nymphal cerci and external wing pads (Fig 8C). *BgKr-h1i+BgE93i* N6 nymphs kept molting into supernumerary nymphal stages until reaching N10 when they arrested development due to problems in shedding the exuvia. The inability to undergo metamorphosis of *BgKr-h1i+BgE93i* nymphs is consistent with our recent observation [33], where RNAi-mediated depletion of *BgE93* in nymphs of *B*. *germanica* prevented the nymphal-adult transition and caused endless reiterations of nymphal molts. Notably, *BgBr-C* mRNA levels in *BgKr-h1i+BgE93i* N6 nymphs were similar to *Control* nymphs (Fig 8E), confirming that the high levels of *BgE93* in *BgKr-h1i* N5 nymphs are responsible for the down-regulation of *BgBr-C* expression.

**Fig 9:**
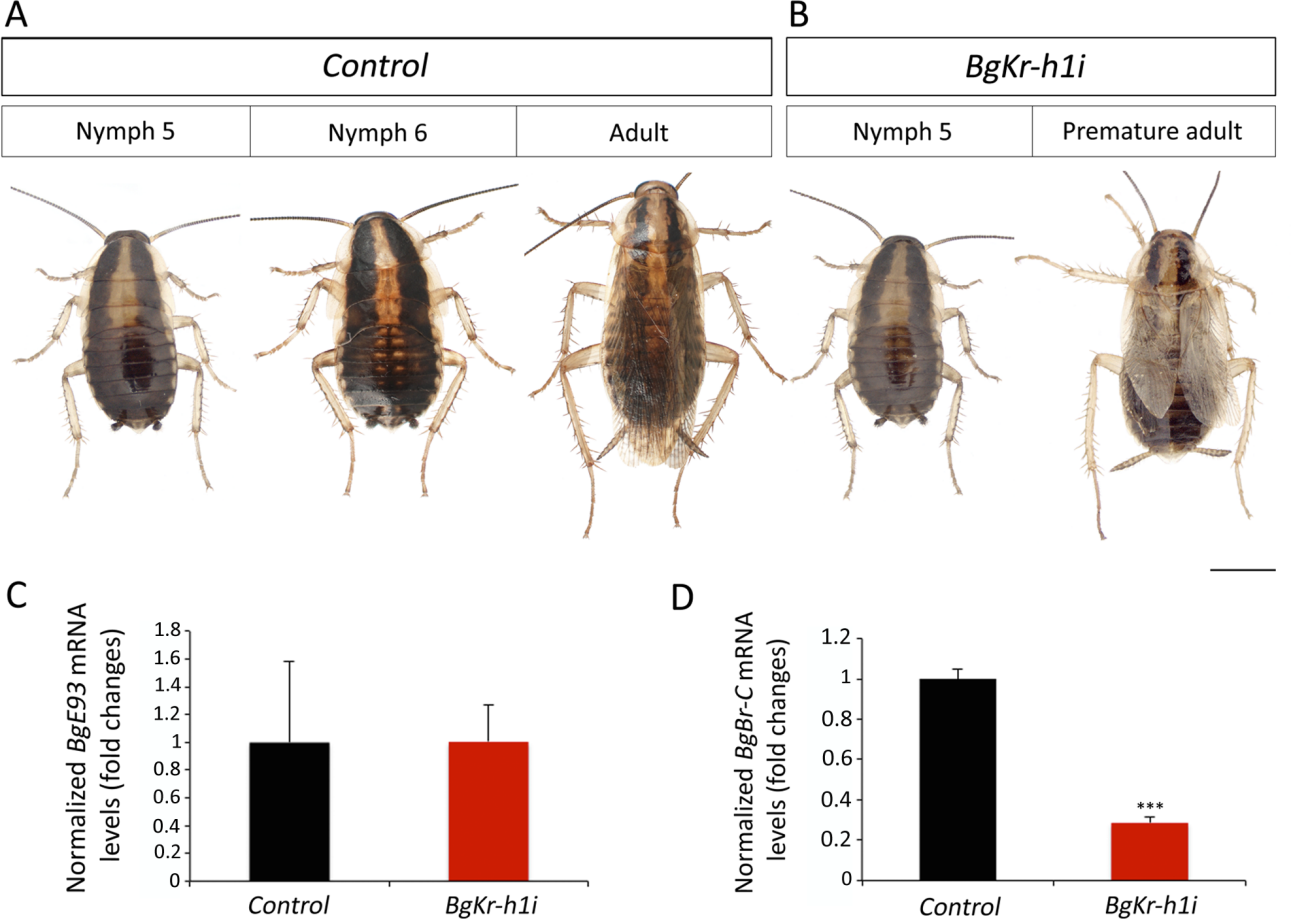
The anti-metamorphic activity of BgKr-h1 is stage-specific. (*A* and *B*) Newly molted N4 nymphs of *B*. *germanica* were injected with *dsMock* (*Control*), or with *dsBgKr-h1* (*BgKr-h1i*) and left until the next molts. (*A*) Dorsal views of *Control* animals after molting into normal N5 and N6 nymphs, and finally to a winged adult. (*B*) Dorsal views of *BgKr-h1i* animals after molting into normal N5 nymphs and then to a premature adult with functional hindwings and forewings, adult-specific pigmentation of the cuticle and the pronotum, and adult cerci. (*C* and *D*) Loss of *BgKr-h1* in N4 does not induce precocious up-regulation of *BgE93*, but represses *BgBr-C* expression. Transcript levels of (*C*) *BgE93*, and (*D*) *BgBr-C* were measured by qRT-PCR in wings from 4-day-old *Control* and *BgKr-h1i* N4 nymphs. Transcript abundance values are normalized against the *BgActin5C* transcript. Fold changes are relative to the expression of each gene in *Control* nymphs, arbitrarily set to 1. Error bars indicate the SEM (n = 5–10). Asterisks indicate differences statistically significant at p≤0.0005 (***) (*t*-test). Scale bar represents 2 mm.

Finally, we aimed to determine whether the BgKr-h1-dependent repression of *BgE93* observed in N5 nymphs is also present in younger nymphal instars. To do this, we injected newly eclosed N4 nymphs with *BgKr-h1* dsRNA and assessed the expression of *BgE93* in these animals. As shown in Fig 9C, *BgE93* mRNA levels were not up-regulated in *BgKr-h1i* N4 nymphs. Consequently, all *BgKr-h1i* N4 nymphs molted properly into normal N5 nymphs (Fig 9A and 9B, S5 Table) and became premature adults in the ensuing molt instead of molting into N6 nymphs as the *Control* animals, which is quite consistent with previous observations [19]. Interestingly, *BgBr-C* mRNA levels in *BgKr-h1i* N4 nymphs were significantly reduced (Fig 9D) despite *BgE93* not being up-regulated in these animals, indicating that BgKr-h1 is necessary to maintain *BgBr-C* expression during the antepenultimate N4 stage, which correlates with previous studies that show that JH enhances *Br-C* expression in hemimetabolous insects [18,24,25]. Overall, these data show that 1) the anti-metamorphic effect of BgKr-h1 during the penultimate N5 instar is channeled through the repression of *BgE93*, suggesting that the metamorphic landscape of hemimetabolous insects has offered the substrate for the evolution of complete metamorphosis and the occurrence of the holometabolan pupa; and 2) the capacity of BgKr-h1 to prevent *BgE93* up-regulation and, hence, premature adult differentiation in *B***.** *germanica* is restricted to the penultimate N5 nymphal stage.

## Discussion

**Fig 10:**
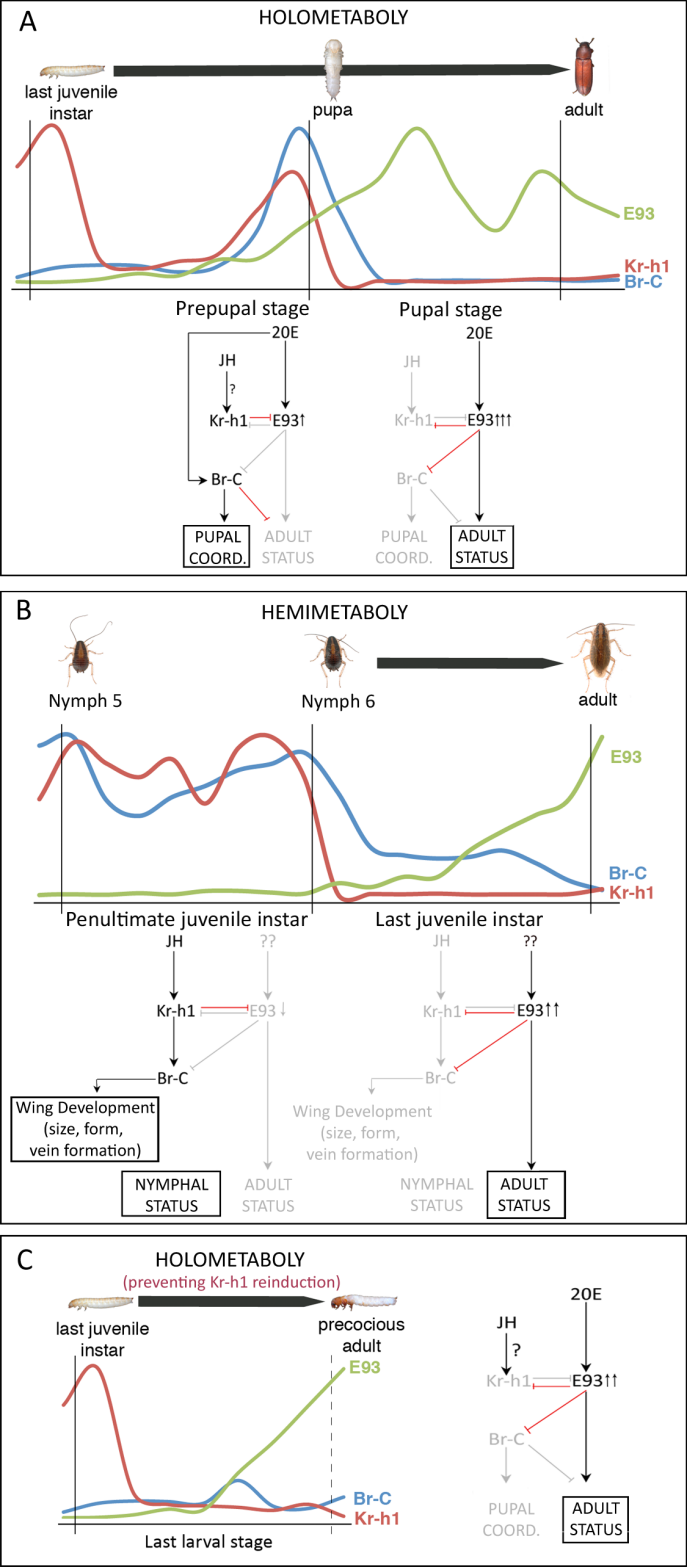
Regulation of holometabolan and hemimetabolan metamorphosis. (*A*) Expression profiles of *Kr-h1*, *E93* and *Br-C* during the last larval and pupal stages (upper part) are from *T*. *castaneum*. Model depicting the regulatory interactions between the metamorphic toolkit genes in the prepupal and pupal stages that underlie the formation of the pupa and the adult using *T*. *castaneum* as a holometabolan model (lower part). Black arrows represent inductive effects, and red lines represent repressive effects. Gray colors denote genes and transcriptional regulatory events that are absent during each particular period. (*B*) Equivalent model depicting the regulation of hemimetabolan metamorphosis during the penultimate and last nymphal instar of *B*. *germanica* as a model. The expression profiles of *Kr-h1*, *E93* and *Br-C* are from *B*. *germanica* [19,25,33]. (C) In the absence of the prepupal *TcKr-h1* peak, the expression dynamics and regulatory interactions of the metamorphic toolkit genes during the last larval instar of *T*. *castaneum* closely resemble those in the last nymphal instar of the hemimetabolous *B*. *germanica*. Question marks denote unknown identities or functional relations. Thick black arrows above the expression profiles represent metamorphic periods.

How complete metamorphosis is controlled at the molecular level is a critical question towards understanding how Holometaboly has evolved from hemimetabolan ancestors. Previous studies have revealed that metamorphosis in both types of insects requires the down-regulation of the anti-metamorphic *Kr-h1* and the up-regulation of the adult specifier *E93* transcription factor genes [18–20,33]. However, a difference arises in holometabolous insects with the transient pulse of *Kr-h1* at the end of the final larval stage, absent in hemimetabolous nymphs. Based on our data, we propose a model through which this late peak of *Kr-h1* might allow the appearance of the new holometabolan-specific pupal stage (Fig 10A). This model is based on the TcKr-h1-dependent repression of *TcE93* expression once metamorphosis has been initiated in the prepupal period (Fig 10A). We propose that the low levels of *TcE93* at this particular stage of development are essential for two reasons; first, it prevents the direct transformation of the larva into the adult, as it happens in the last nymphal instar of hemimetabolous insects. Second, it allows the stage-specific burst of *TcBr-C* expression in the prepupal stage, as TcE93 is a potent repressor of *TcBr-C* expression (Figs 3 and 7; [33]). This pulse of *TcBr-C* is crucial to coordinate the morphogenesis of the different body parts that would give rise to the normal pupal morphology, as evidenced by the occurrence of larva-pupa-adult mosaics in *TcBr-C*-depleted animals [26–28]. Thus, as a result of the crosstalk between *TcKr-h1*, *TcE93* and *TcBr-C* in the prepupal stage, the larva molts into a new metamorphic stage, the pupa, instead of undergoing the terminal adult molt. Finally, our previous results showed that after the pupal molt, high levels of *TcE93* repress *TcKr-h1* and *TcBr-C* expression, ensuring the completion of the metamorphic process (Fig 10A) [33].

From an evolutionary perspective, our results strongly suggest that the metamorphic genetic landscape of hemimetabolous insects has served as the substrate for the evolution of complete metamorphosis. First, RNAi analysis in the hemimetabolous insect *B*. *germanica* (Figs 8 and 9), coupled with previous results [33], reveals the fundamental conservation of the functional interactions between the metamorphic network genes *Kr-h1*, *E93* and *Br-C* in hemimetabolous and holometabolous insects (Fig 10B). Second, in the absence of the prepupal pulse of *TcKr-h1* (*TcKr-h1i* animals), the expression dynamics of the metamorphic network genes during the last larval instar of *T*. *castaneum* closely resemble those in the last nymphal instar of hemimetabolous insects (Fig 10C). Therefore, it seems likely that the transient re-induction of *Kr-h1* midway through the metamorphic process in holometabolous ancestors, with the consequent redeployment of the metamorphic toolkit circuit, occurred in the origin of the pupal stage, transforming the single-stage metamorphic period of hemimetabolous insects into the two-stages process of holometabolous insects. Consistent with this scenario, the expression profile of *Kr-h1* during the post-embryonic development in thrips (Thysanoptera), one of the closest hemimetabolous relatives of holometabolous insects that present quiescent and non-feeding stages called propupa and pupa (Neometaboly), are comparable to those in holometabolous insects [39]. Based on our results, we thus propose that the two metamorphic periods of holometabolous insects, the last larval instar and the pupal period, are ontogenetically homologous to the last nymphal instar of hemimetabolous insects.

Although the regulatory architecture between the metamorphic toolkit genes is mostly conserved in winged insects, as are the metamorphic functions of Kr-h1 and E93, the specific role of Br-C in relation to metamorphosis has dramatically changed during the evolution of holometaboly. While Br-C does not exert any metamorphic role in hemimetabolous insects, and its function is mainly limited to the control of wing development, particularly in relation to size, form and vein formation [18,24,25], in holometabolous insects Br-C has been specifically recruited for new stage-specific metamorphic functions [26–29,31,32]. Therefore, Br-C acts as the pupal coordinator that ensures proper pupal morphogenesis and prevents premature adult morphogenesis in the holometabolan context (Fig 5). The acquisition by Br-C of new metamorphic functions has been favored, in part, by changes in its expression, from being constantly expressed during embryogenesis and throughout nymphal development in Hemimetaboly to be confined to the strong prepupal-specific pulse characteristic of holometabolous insects [20,26,27,40–42]. Two events have probably facilitated this change. First, the conserved repressive activity of E93 upon *Br-C* expression, already present in hemimetabolous insects, that ensured the repression of *Br-C* during the pupal stage [33]. Second, a shift in the JH regulatory activity on *Br-C* expression, from being inductive in hemimetabolous insects [18,24,25] to repressive in holometabolous insects [40,43,44]. The JH-dependent repression of *Br-C* has restricted the expression of this factor in young larvae until the onset of the last larval instar, when the decline of JH and the temporal disappearance of *Kr-h1* allow the induction of *Br-C* by the ecdysteroid hormone 20-hydroxyecdysone (20E) [40,41,45]. Recently, it has been shown in *B*. *mori* that the early larval repression of *Br-C* depends on the direct binding of Kr-h1 to the *Br-C* gene [46]. Paradoxically, the repressive activity of Kr-h1 on *Br-C* expression does not occur during the prepupal stage of holometabolous insects as the strong prepupal pulse of *Br-C* parallels that of *Kr-h1*. Consistent with this observation, the overexpression of *BmKr-h1* during the prepupal stage in transgenic *B*. *mori* cannot prevent the normal appearance of the *BmBr-C* pulse [47]. According to that, our data demonstrate that the prepupal surge of *Kr-h*1 is required to allow the normal expression of *Br-C* through the repression of *E93*, which suggests that the regulatory activity of Kr-h1 upon *Br-C* expression is stage-specific, from directly inhibiting its expression in young larvae to allowing its induction in the prepupal stage through the repression of E93. Further studies are needed to reveal the molecular mechanisms through which *Br-C* is not repressed by Kr-h1 in the prepupal stage.

Given the functional relevance of the reappearance of Kr-h1 during the prepupal period, it remains to be established what is the precise signal that controls it. As *Kr-h1* expression is induced by JH in hemimetabolous and holometabolous species [18–20,48–51], it is plausible that a prepupal pulse of circulating JH controls *TcKr-h1* upregulation. Consistent with this possibility, allatectomy (the surgical elimination of the gland that synthesizes JH, the *corpora allata*) in the final larval instar of the lepidopterans *Manduca sexta* and *Hyalophora cecropia* caused partial premature adult development [52,53]. However, the double knockdown of *TcJHAMT* and *TcCYP15A1*, the two enzymes that catalyze the final two steps of the JH biosynthetic pathway in *T*. *castaneum*, does not cause precocious differentiation of adult structures [54]. Likewise, allatectomized *Bombyx mori* larvae developed into normal pupa, which indicates that the prepupal *BmKr-h1* pulse is *corpora allata*-independent [47]. As *BmKr-h1* expression *in vitro* is up-regulated by JH, it is possible that *BmKr-h1* could be induced by JH synthesized from other tissues than the *corpora allata* [47]. Unlike lepidopteran species, *Kr-h1* expression in *D*. *melanogaster* is not only induced by JH but also by 20E [51,55]. Overall, these data suggest that the critical prepupal up-regulation of *Kr-h1* in holometabolous insects is controlled by a combination of factors, including JH and 20E, still to be clearly identified.

On the other hand, it is of great interest to know how *E93* expression is regulated. While the repressive activity of Kr-h1 on *E93* expression is a common trait of hemimetabolous and holometabolous insects (Figs 3 and 8; [38]), the signals that induce *E9*3 expression have only been characterized in two holometabolous insects, *D*. *melanogaster* and *B*. *mori*. In the fly, *DmE93* expression is induced by 20E [56,57]. Likewise, in *B*. *mori*, it has been shown that *BmE93* is also induced by 20E and repressed by JH [58]. Given the relevance of such regulation, future studies should investigate the molecular basis underlying the regulation of E93 expression in hemimetabolous and holometabolous insects.

In conclusion, we have established the critical stage-specific interactions between the metamorphic toolkit genes that underlie the formation of the pupa in holometabolous insects. Although the full details of the origin of the holometabolan pupa still remain to be determined, our results provide a molecular framework to explain how complete metamorphosis is regulated, thus shedding light into the evolution of complete metamorphosis.

## Materials and Methods

### Insects

Wild-type *T*. *castaneum* strain and the enhancer-trap line *pu11* (obtained from Y. Tomoyasu, Miami University, Oxford, Ohio) were reared on organic wheat flour containing 5% nutritional yeast, and maintained at 29°C in constant darkness. Flies were raised on standard *D*. *melanogaster* medium at 25°C, unless otherwise required. Oregon R flies (*OR-R*, used as a wild type control), *ActGAL4*, *rnGal4*, *CiGAL4*, *UASGFP* and *UASdicer* (used to enhance RNAi effectiveness) were obtained from the Bloomington Stock Center (BDSC). *UAS-DE93*^*RNAi*^ (KK108140; GD4449), and *UAS-DKr-h1*^*RNAi*^ (KK107935; GD51282) are from the Vienna *Drosophila* RNAi Center (VDCR). For clonal analysis, *hsflp;Tub>y>Gal4;UASGFP* females were crossed with males carrying *UASKr-h1*^*RNAi*^. Embryos were kept at 25°C until late L2, incubated 1 hour at 37°C and transferred to 25°C until late L3. *B*. *germanica* specimens were reared in the dark at 30 **±** 1°C and 60–70% relative humidity.

### Quantitative real-time reverse transcriptase polymerase chain reaction (qRT-PCR)

Total RNA was isolated with the GenElute Mammalian Total RNA kit (Sigma), DNAse treated (Promega) and reverse transcribed with Superscript II reverse transcriptase (Invitrogen) and random hexamers (Promega). To obtain cDNA from the wing pouch of *rnGal4* and *rnGal4>UASDmKr-h1*^*RNAi*^ animals, the wing pouch was specifically separated from the rest of the wing. Relative transcripts levels were determined by real-time PCR (qPCR), using Power SYBR Green PCR Mastermix (Applied Biosystems). To standardize the qPCR inputs, a master mix that contained Power SYBR Green PCR Mastermix and forward and reverse primers was prepared (final concentration: 100nM/qPCR). The qPCR experiments were conducted with the same quantity of tissue equivalent input for all treatments and each sample was run in duplicate using 2 μl of cDNA per reaction. All the samples were analyzed on the iCycler iQ Real Time PCR Detection System (Bio-Rad). For each standard curve, one reference DNA sample was diluted serially. Primers sequences for qPCR analyses were:

*T*. *castaneum*:

*TcE93*: TcE93-F: 5’-CTCTCGAAAACTCGGTTCTAAACA-3’

TcE93-R: 5’-TTTGGGTTTGGGTGCTGCCGAATT-3’

*TcBr-C*: TcBr-C-F: 5’-TCGTTTCTCAAGACGGCTGAAGTG-3’

TcBr-C-R: 5’-CTCCACTAACTTCTCGGTGAAGCT-3’

*TcKr-h1*: TcKr-h1-F: 5’-AAGAAGAGCATGGAAGCACACATT-3’

TcKr-h1-R: 5’-GAATCGTAGCTAAGAGGGTCTTGA-3’

*TcCPR27*: TcCPR27-F: 5’-AGGTTACGGCCATCATCACTTGGA-3’

TcCPR27-R: 5’-ATTGGTGGTGGAAGTCATGGGTGT-3’

*TcRpL32*: TcRpL32-F: 5’-CAGGCACCAGTCTGACCGTTATG-3’

TcRpL32-R: 5’-CATGTGCTTCGTTTTGGCATTGGA-3’

*D*. *melanogaster*:

*DmE93A*: DmE93A-F: 5’-CACATCAGCAGCTATGAAATA-3'

mDE93A-R: 5’- AACCGGCTATTGCTATGGGCTGTT-3'

*DmE93B*: DmE93B-F: 5’-TCCACAGATATGCTGCATATTGTG-3'

DmE93A-R: 5’- AACCGGCTATTGCTATGGGCTGTT-3'

*DmBr-C*: DmBr-C-F: 5’-CATCTGGCTCAGATACAGAACCT-3’

DmBr-C-R: 5’-CTTCAGCAGCTGGTTGTTGATGT-3’

*DmRpL32*: DmRpL32-F: 5’-CAAGAAGTTCCTGGTGCACAA-3’

DmRpL32-R: 5’-AAACGCGGTTCTGCATGAG-3’

*B*. *germanica*:

*BgE93*: BgE93-F: 5’-CAAGCGGGGCAAATATCGCAATTA-3’

BgE93-R: 5’-TGACCTTGTACTCGAGTGTGG-3’

*BgBr-C*: BgBr-C-F: 5’-CTTAAAGCTCATAGAGTGGTGTTG-3’

BgBr-C-R: 5’-CACTTCACCATGGTATATGAATTC-3’

### RNA interference (RNAi)

*T*. *castaneum—*RNAi *in vivo* was performed as previously described [33,59]. Control dsRNA consisted of a non-coding sequence from the pSTBlue-1 vector (*dsControl*). For the *in vivo* treatment, dsRNAs, concentrated up to 4 µg/µl, were injected into the abdomen of last instar larvae (L7) of the *pu11* line. *B*. *germanica*–RNAi *in vivo* was performed as previously described [60,61]. A dose of 1 µl (5–8 µg/µl) of the dsRNA solution was injected into the abdomen of newly ecdysed penultimate (N5) or antepenultimate (N4) instar nymphs, and left until analysed. In case of coinjection of two dsRNAs in *T*. *castaneum* or *B*. *germanica*, the same volume of each dsRNA solution was mixed and applied in a single injection. To maintain the RNAi effect during the successive nymphal instars, the same dose of dsRNAs was reapplied to all treated animals after molting into new nymphal stages. The primers used to generate templates via PCR for transcription of the dsRNAs were:

*T*. *castaneum*:

*dsTcKr-h1*: dsTcKr-h1-F: 5’**‐** AATCCTCCTGCTCATCCAGCACTA-3’

dsTcKr-h1-R: 5’**‐** CAGGATTCGAACTAGGAGGTGTTA-3’

*dsTcE93*: dsTcE93-F: 5’-AAATAACGGTGATACAGTGTCAAG-3’

dsTcE93-R: 5’-TTGTAGTCCATCTCGGAGATGGAA-3’

*dsTcBr-C*: dsTcBr-C-F: 5’-CAATTACCAAAGCAGCATCACATC-3’

dsTcBr-C-R: 5’-GGCTTTGTACTTGCGCCAACTGTT-3’

*B*. *germanica*:

*dsBgKr-h1*: dsBgKr-h1-F: 5’- GAATCTCAGTGTGCATAGGCG-3’

dsBgKr-h1-R: 5’- CCTTGCCACAAATGACACAA-3’

*dsBgE93*: dsBgE93-F: 5’-GAAACAGAACCTCCTTTCAAAAGG-3’

dsBgE93-R: 5’-AAAGTGTGAACCTGCCCGATGAA-3’

### Microscopy, histological analysis and immunocytochemistry

*T*. *castaneum* dissections were carried out in Ringer’s saline and the different appendages were mounted directly in Glycerol 70%. For *D*. *melanogaster* immunohistochemistry, wings from 0 h after puparium formation animals were collected and stained as described [33]. Antibodies: mouse anti-Broad-Complex (1:100; Developmental Studies Hybridoma Bank (DSHB)); rat anti-Spalt (Sal) (1:200; a gift from R. Barrio); rabbit anti-Caspase 3 (1:250; Cell Signaling Technologies); Alexa Fluor 555-conjugated secondary antibodies (1:200; Molecular Probes). All samples were examined with AxioImager.Z1 (ApoTome 213 System, Zeiss) microscope, and images were subsequently processed using Adobe photoshop.

### Scanning-electron microscopy

*Control* and *TcE93i* animals of *T*. *castaneum* were carefully taken out of the larval cuticle with forceps when necessary. Then, they were fixed in 80% ethanol, and dehydrated with a series of graded ethanol solutions (90%, 95% and 100%) for 15 min in each solution, critical-point dried using CO_2_, sputter-coated with gold-palladium, and observed under a Hitachi S-3500N scanning electron microscope.

## Supporting Information

S1 Fig Developmental expression profiles of the metamorphic genetic network genes *TcE93*, *TcKr-h1* and *TcBr-C* in the last larval instar (L7) and the pupal period of the holometabolous insect *T*. *castaneum*. *TcKr-h1* mRNA levels were measured by qRT-PCR. Transcript abundance values are normalized against the *TcRpL32* transcript. Fold changes are relative to the expression of *TcKr-h1* in newly emerged L7 larvae, arbitrarily set to 1. Error bars indicate the SEM (n = 5). Data on *TcE93* and *TcBr-C* levels are from (24).
(PDF)

S2 Fig The abdomen of *TcKr-h1*-depleted animals shows several pupal features. Comparison of abdominal features in (*A* and *E*) *Control* pupa, (*B* and *F*) *Control* adult, and (*C*, *D* and *G*, *H*) *TcKr-h1i* animals, showing the presence of short urogomphi (arrows in *A-D*) and malformed gin traps (arrows in *E-H*). Scale bars represent 100 µm in (*A-C*), (*E*) and (*G*); 0.5 mm in (*D*) and (*H*); 50 µm in (*F*).
(PDF)

S3 Fig Pupal phenotypes obtained from *TcBr-C*-depleted *T*. *castaneum* larvae. Newly molted L7 larvae were injected with *dsMock* (*Control*) or with *dsTcBr-C* (*TcBr-Ci*). (*A*-*F’*) Comparison of the external morphology of appendages between (*A-F*) *Control*, and (*A’-F’*) *TcBr-Ci* animals after the pupal molt. *TcBr-Ci* pupae show abnormal differentiation of pupal characters such as (*A* and *A’*) urogomphi (arrows), and (*B* and *B’*) gin traps (arrow) in the abdomen, as well as accelerated adultization of thoracic and cephalic appendages, such as (*C* and *C’*) legs, (*D* and *D’*) antennae, (*E* and *E’*) maxilla, and (*F* and *F’*) mandible. The appendages of *TcBr-Ci* pupae presented premature segmentation but larval-like pigmentation.
(PDF)

S4 Fig Loss of *TcKr-h1* in last instar larvae does not induce apoptosis in *T*. *castaneum* wings. (*A* and *B*) Caspase-3 and DAPI stainings in wings of (A) *Control* and (B) *TcKr-h1i* prepupa. Depletion of *TcKr-h1* does not increase the number of Caspase-3 positive cells.
(PDF)

S1 Table Phenotypes of *T*. *castaneum* injected with *dsTcKr-h1* in the last larval instar. (DOCX)

S2 Table Phenotypes of *T*. *castaneum* injected with *dsTcKr-h1* and *dsTcE93* simultaneously in the last larval instar. (DOCX)

S3 Table Phenotypes of *T*. *castaneum* injected with *dsTcBr-C* in the last larval instar. (DOCX)

S4 Table Phenotypes of *B*. *germanica* injected with different *dsRNAs* in the penultimate (N5) nymphal instar. (DOCX)

S5 Table Phenotypes of *B*. *germanica* injected with different *dsRNAs* in the antepenultimate (N4) nymphal instar. (DOCX)
