# Supplementary figures and images for: The Occurrence of the Holometabolous Pupal Stage Requires the Interaction between E93, Krüppel-Homolog 1 and Broad-Complex
Source: PLoS Genet. 2016 May 2;12(5):e1006020. doi: 10.1371/journal.pgen.1006020 (PMC4852927; doi:10.1371/journal.pgen.1006020)

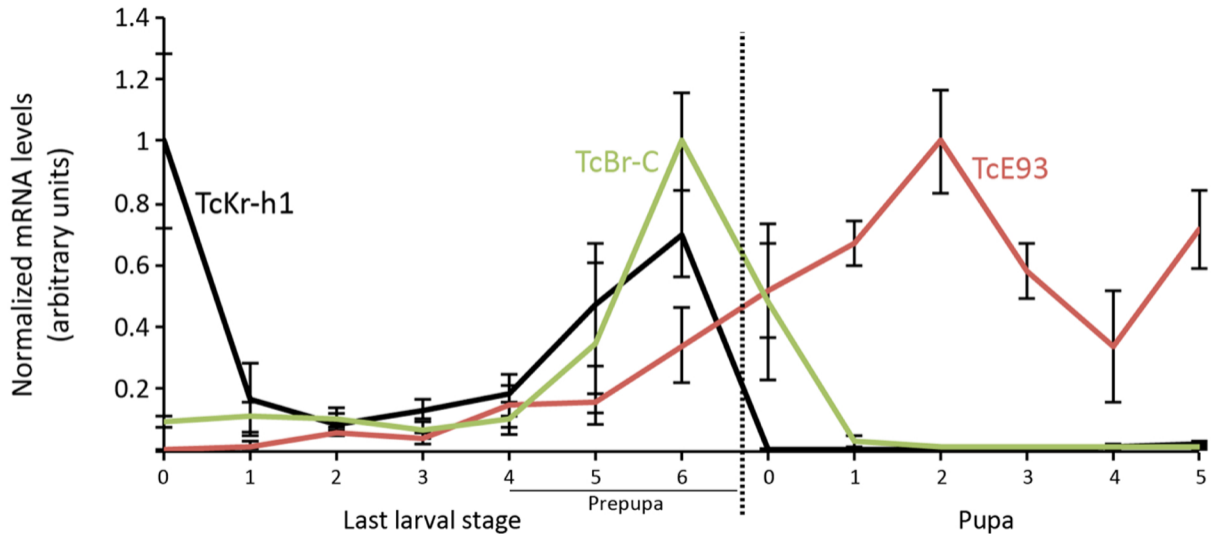

Supplement: S1 Fig — TcKr-h1 mRNA levels were measured by qRT-PCR. Transcript abundance values are normalized against the TcRpL32 transcript. Fold changes are relative to the expression of TcKr-h1 in newly emerged L7 larvae, arbitrarily set to 1. Error bars indicate the SEM (n = 5). Data on TcE93 and TcBr-C levels are from (24). (PDF) [file pgen.1006020.s001.pdf]

Control

Pupa

Adult

TcKr-h1i

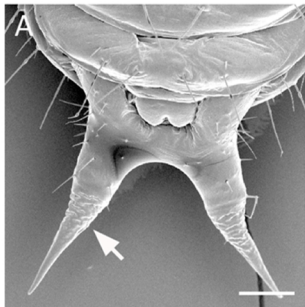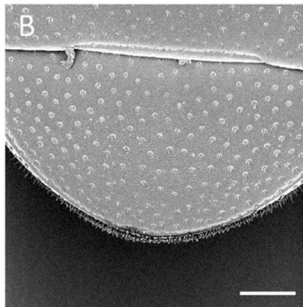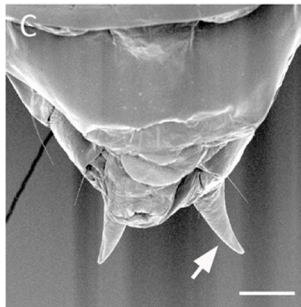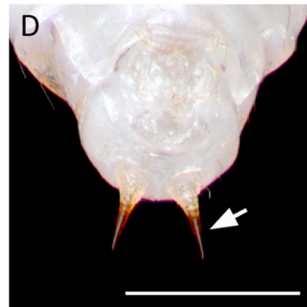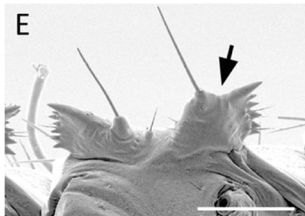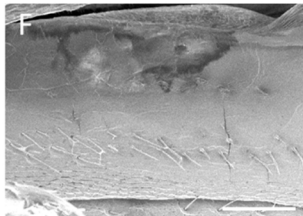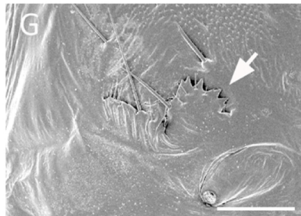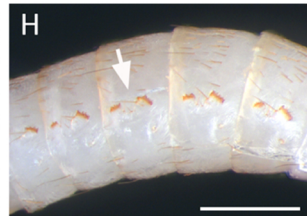

Supplement: S2 Fig — Comparison of abdominal features in (A and E) Control pupa, (B and F) Control adult, and (C, D and G, H) TcKr-h1i animals, showing the presence of short urogomphi (arrows in A-D) and malformed gin traps (arrows in E-H). Scale bars represent 100 µm in (A-C), (E) and (G); 0.5 mm in (D) and (H); 50 µm in (F). (PDF) [file pgen.1006020.s002.pdf]

Control

TcBri

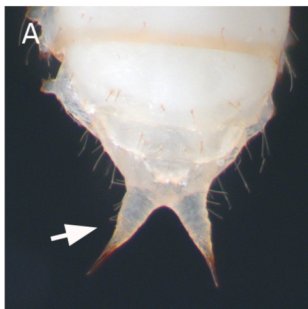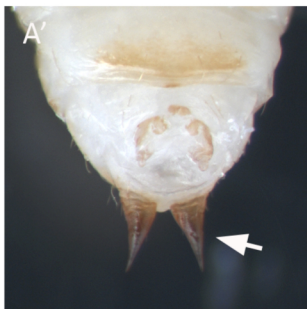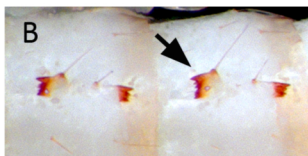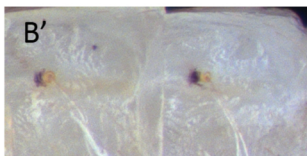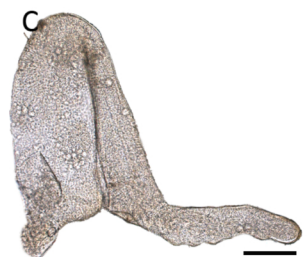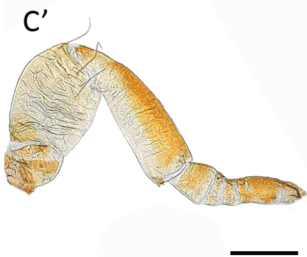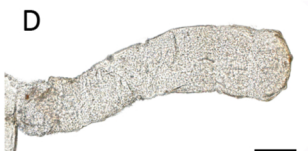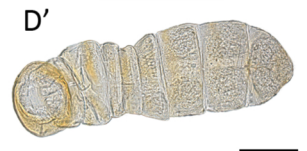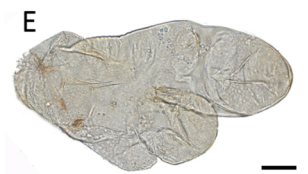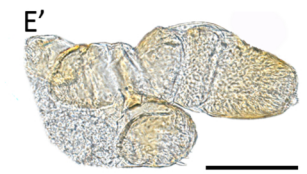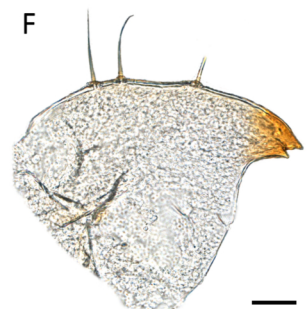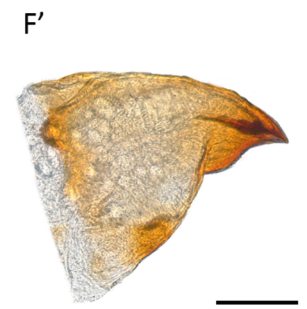

Supplement: S3 Fig — Newly molted L7 larvae were injected with dsMock (Control) or with dsTcBr-C (TcBr-Ci). (A-F’) Comparison of the external morphology of appendages between (A-F) Control, and (A’-F’) TcBr-Ci animals after the pupal molt. TcBr-Ci pupae show abnormal differentiation of pupal characters such as (A and A’) urogomphi (arrows), and (B and B’) gin traps (arrow) in the abdomen, as well as accelerated adultization of thoracic and cephalic appendages, such as (C and C’) legs, (D and D’) antennae, (E and E’) maxilla, and (F and F’) mandible. The appendages of TcBr-Ci pupae presented premature segmentation but larval-like pigmentation. (PDF) [file pgen.1006020.s003.pdf]

Control

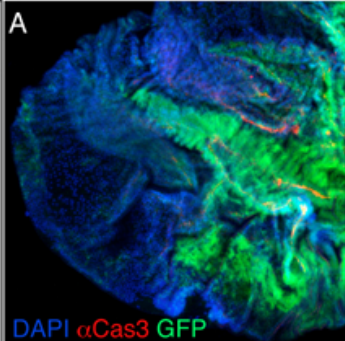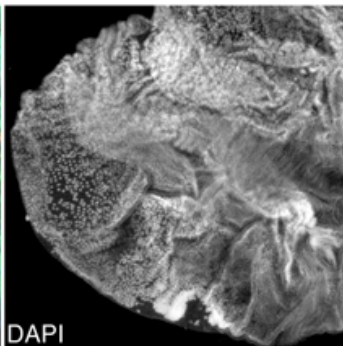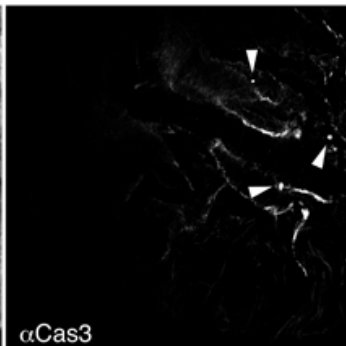

TcKr-h1i

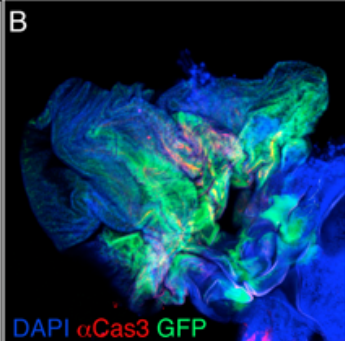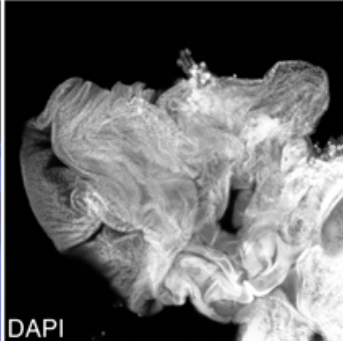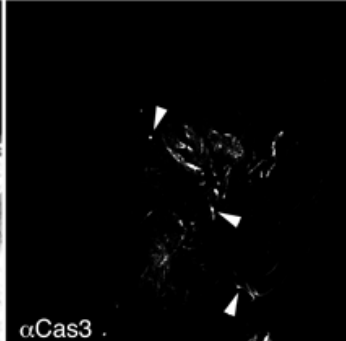

Supplement: S4 Fig — (A and B) Caspase-3 and DAPI stainings in wings of (A) Control and (B) TcKr-h1i prepupa. Depletion of TcKr-h1 does not increase the number of Caspase-3 positive cells. (PDF) [file pgen.1006020.s004.pdf]
