# Supplementary material for: The Occurrence of the Holometabolous Pupal Stage Requires the Interaction between E93, Krüppel-Homolog 1 and Broad-Complex
Source: PLoS Genet. 2016 May 2;12(5):e1006020. doi: 10.1371/journal.pgen.1006020 (PMC4852927; doi:10.1371/journal.pgen.1006020)
Supplement: S1 Table — (DOCX) [file pgen.1006020.s005.docx]

**S1 Table.** Phenotypes of *T. castaneum* injected with *dsTcKr-h1* in the last larval instar.

| Treatment^a^ | n | Larval mortality | Pupa | Prepupal arrest |
| --- | --- | --- | --- | --- |
| *Control* | 90 | 12 (13.3 %) | **78 (86.7 %)** | ― |
| *TcKr-h1i* | 144 | 18 (12.5 %) | 17 (11.8 %) | **109 (75.7 %)** |

^a^ The *dsRNAs* are injected in last instar larvae (L7), and the phenotypes are scored on the larval-pupal transition.
