# Supplementary material for: The Occurrence of the Holometabolous Pupal Stage Requires the Interaction between E93, Krüppel-Homolog 1 and Broad-Complex
Source: PLoS Genet. 2016 May 2;12(5):e1006020. doi: 10.1371/journal.pgen.1006020 (PMC4852927; doi:10.1371/journal.pgen.1006020)
Supplement: S2 Table — (DOCX) [file pgen.1006020.s006.docx]

**S2 Table.** Phenotypes of *T. castaneum* injected with *dsTcKr-h1* and *dsTcE93* simultaneously in the last larval instar.

| Treatment^a^ | n | Larval mortality | Pupa | Prepupal arrest | |
| --- | --- | --- | --- | --- | --- |
|  |  |  |  | Pupal-like | Adult-like |
| *Control* | 47 | 9 (19.1 %) | **38 (80.9 %)** | ― | ― |
| *TcKr-h1i*  *+*  *TcE93i* | 110 | 18 (16.4 %) | **45 (40.9 %)** | **46 (41.8 %)** | 1 (0.9 %) |

^a^ The *dsRNAs* are injected in last instar larvae (L7), and the phenotypes are scored on the larval-pupal transition.
