# Supplementary material for: The Occurrence of the Holometabolous Pupal Stage Requires the Interaction between E93, Krüppel-Homolog 1 and Broad-Complex
Source: PLoS Genet. 2016 May 2;12(5):e1006020. doi: 10.1371/journal.pgen.1006020 (PMC4852927; doi:10.1371/journal.pgen.1006020)
Supplement: S3 Table — (DOCX) [file pgen.1006020.s007.docx]

**S3 Table.** Phenotypes of *T. castaneum* injected with *dsTcBr-C* in the last larval instar**.**

| Treatment^a^ | n | Larval mortality | Pupa | Pupal phenotype | |
| --- | --- | --- | --- | --- | --- |
|  |  |  |  | Prepupal arrest | Pupal  molt^b^ |
| *Control* | 93 | 7 (7.5 %) | **86 (92.5 %)** | ― | ― |
| *TcBr-Ci* | 106 | 5 (4.7 %) | ― | **46 (43.4 %)** | **55 (51.9 %)** |

^a^ The *dsRNAs* are injected in last instar larvae (L7), and the phenotypes are scored on the larval-pupal transition.

^b^These animals show a mix of larval, pupal and adult characters
