# Supplementary material for: The Occurrence of the Holometabolous Pupal Stage Requires the Interaction between E93, Krüppel-Homolog 1 and Broad-Complex
Source: PLoS Genet. 2016 May 2;12(5):e1006020. doi: 10.1371/journal.pgen.1006020 (PMC4852927; doi:10.1371/journal.pgen.1006020)
Supplement: S4 Table — (DOCX) [file pgen.1006020.s008.docx]

**S4 Table.** Phenotypes of *B. germanica* injected with different *dsRNAs* in the penultimate (N5) nymphal instar.

| Treatment^a^ | n | Nymphal  mortality | Precocious adult | Normal  Nymph (N6) |
| --- | --- | --- | --- | --- |
| *Control* | 56 | 0 (0 %) | 0 (0 %) | **56** **(100 %)** |
| *BgKr-h1i* | 45 | 0 (0 %) | **40** **(89 %)** | 5 (11 %) |
| *BgKr-h1i*  *+*  *BgE93i* | 38 | 3 (8 %) | 0 (0 %) | **35 (92 %)** |

^a^ The *dsRNAs* are injected in newly molted N5 nymphs, and the phenotypes are scored after one molt.
