# Supplementary material for: The Occurrence of the Holometabolous Pupal Stage Requires the Interaction between E93, Krüppel-Homolog 1 and Broad-Complex
Source: PLoS Genet. 2016 May 2;12(5):e1006020. doi: 10.1371/journal.pgen.1006020 (PMC4852927; doi:10.1371/journal.pgen.1006020)
Supplement: S5 Table — (DOCX) [file pgen.1006020.s009.docx]

**S5 Table.** Phenotypes of *B. germanica* injected with different *dsRNAs* in the antepenultimate (N4) nymphal instar.

| Treatment^a^ | n | Nymphal  mortality | Normal  Nymph (N5) | Normal  Nymph (N6) | Precocious adult |
| --- | --- | --- | --- | --- | --- |
| *Control* | 18 | 0 (0 %) | **18** **(100 %)** | **18 (100 %)** | 0 (0 %) |
| *BgKr-h1i* | 20 | 0 (0 %) | 20 (100 %) | 3 (15 %) | **17 (85 %)** |

^a^ The *dsRNAs* are injected in newly molted N4 nymphs, and the phenotypes are scored after the successive molts.
